# *OsRDH* modulates multiple grain quality traits and contributes to rice seed germination and root growth development

**DOI:** 10.3389/fpls.2026.1820692

**Published:** 2026-05-20

**Authors:** Bo Peng, Jing Qiu, Ziyue Huang, Qiaoyu Zhang, Qiang Zhao, Zhiguo Zhang, Yujian Wang, Yaqin Huang, Wei Zhou, Jinhui Zhao, Guanwang Shen, Yuliang Qi, Yanfang Sun, Quanxiu Wang

**Affiliations:** 1 College of Life Sciences, Xinyang Normal University, Xinyang, China; 2 College of Horticulture, Xinyang Agriculture and Forestry University, Xinyang, China; 3 Henan Scientific Research Platform Service Center, Zhengzhou, China; 4 Henan Lingrui Pharmaceutical Compan y Limited, Xinyang, China; 5 College of Pharmacy, Xinyang Agriculture and Forestry University, Xinyang, China; 6 Xinyang Academy of Agricultural Science, Xinyang, China

**Keywords:** grain quality, *Osrdh*, rice, root growth development, seed germination

## Abstract

Retinol dehydrogenase (RDH) catalyzes key steps in retinol-associated metabolic pathways in animals and has been implicated in multiple physiological processes in plants. Despite this, the contribution of the rice RDH gene (*OsRDH*) to plant development and grain quality formation has not been clearly defined. In earlier work, the important quality trait gene *OsGAPC3* was isolated and cloned from rice. Through an in-depth investigation of the molecular regulation mechanisms underlying *OsGAPC3*, transcriptomic sequencing analysis revealed a marked upregulation of *OsRDH* expression in the *Osgapc3* mutant. Subsequent studies demonstrated that *OsRDH* is constitutively expressed in rice, with its encoded protein localized in the chloroplasts. Loss-of-function mutation of *OsRDH* resulted in altered endosperm storage organization: protein bodies in mutant grains were reduced in size, accompanied by substantial decreases in total protein accumulation and essential amino acid content, ultimately diminishing grain nutritional value. In contrast, grain morphology was favorably modified, with elongated grains and pronounced reductions in both the extent and severity of endosperm chalkiness. These changes were associated with a more compact arrangement of starch granules, leading to improved grain appearance. Notably, the amylose content in *Osrdh* mutant rice was significantly lower, while milled rice yield was significantly higher, contributing to improvements in both processing quality and cooking quality. At the physiological level, disruption of *OsRDH* perturbed the transcription of genes involved in gibberellin (GA) and abscisic acid (ABA) metabolic pathways, thereby influencing seed germination behavior, root growth dynamics. Thus, these results demonstrate that *OsRDH* is an important quality trait gene with multiple biological functions, and is involved in various biological processes such as rice seed germination and root growth and development. Simultaneously, *OsRDH* represents a valuable target for the genetic improvement of grain appearance, processing performance, and cooling/eating quality through molecular breeding strategies.

## 1 Introduction

Globally, rice (*Oryza sativa* L.) represents a fundamental component of human diets, supporting the nutritional demands of over half of the world’s inhabitants and accounting for the principal staple intake of more than two-thirds of the population in China (Li et al., 2022; Zhao et al., 2022). As the economy and society continue to develop and living standards improve, consumers are placing increasingly higher demands on rice quality. Rice quality is a multifaceted concept, encompassing appearance quality, nutritional quality, processing quality, and cooking and eating quality (Peng et al., 2017; Hao et al., 2019). The appearance quality of rice is mainly governed by grain morphology, chalkiness characteristics, and endosperm translucency. In contrast, nutritional quality is determined by the accumulation of essential components, including proteins, starch, fatty acids, and amino acids. Among these constituents, rice protein is recognized as a high-value dietary protein due to its high digestibility and efficient absorption in humans, rendering both its concentration and compositional profile critical parameters for assessing rice nutritional quality (Peng et al., 2017; Pereira et al., 2022). In rice grains, protein represents the second most abundant nutrient fraction following carbohydrates and can be categorized into albumin, globulin, prolamin, and glutelin according to differences in solubility (Peng et al., 2014a; He et al., 2013). Among these, Glutelin is the most digestible protein, comprising approximately 80% of the protein content in the endosperm. Alcohol-soluble proteins account for about 20%, while albumin and globulin make up roughly 5% and 10% of the total protein content, respectively (Shewry and Halford, 2002). The protein concentration in rice grains generally varies from 4.3% to 19.3%, and rice-derived protein contributes approximately 15% of the total protein intake of the global population (He et al., 2013). Processing quality of rice is commonly evaluated using three parameters: brown rice rate, milled rice rate, and head rice rate (Lu et al., 2025; Xia et al., 2021). Cooking and eating quality encompasses the sensory attributes and physicochemical properties exhibited by rice during cooking and consumption (Zhang et al., 2024; Li et al., 2025). These properties are primarily characterized by amylose content, taste value (TV), gel consistency (GC), and gelatinization temperature (GT) (Li et al., 2024; Bao et al., 2023). As a comprehensive indicator of rice quality, cooking and eating quality play a decisive role in consumer preference and purchasing decisions.

In animals, retinol dehydrogenase (RDH) is the core enzyme in vitamin A metabolism, catalyzing the conversion of retinol to retinaldehyde and subsequently to retinoic acid. The generated retinoic acid functions as a signaling molecule that modulates a wide range of biological activities, including energy metabolism, lipid biosynthesis and catabolism, intracellular transport, and protein synthesis and post-translational modification (Wu et al., 2023; Wang et al., 2022; Leung et al., 2024). In plants, RDH isozymes (such as Arabidopsis *AtSDR1*) catalyze the redox reaction of carotenoid derivatives (such as β-ionone) to produce plant-specific retinaldehyde analogs. These analogs regulate reactive oxygen species (ROS) and phytohormone levels by modulating carotenoid derivatives (Cheng et al., 2002). Notably, the plant RDH metabolic network and hormone signaling pathways are intricately cross-regulated. RDH not only regulates ROS levels and antioxidation responses through its metabolites, but it also influences the biosynthesis and signal transduction of ABA and IAA, thereby collaboratively regulating cell growth and differentiation (Carazo et al., 2021; Chang and Zhang, 2005; Xu et al., 2024). As such, RDH is a key enzyme in the metabolism of retinol and vitamin A, playing an important role in processes such as cell growth and differentiation in animals. RDH isozymes in plants exhibit high functional diversity and are involved in various biological processes such as metabolism, development, and stress resistance (Cheng et al., 2002; Carazo et al., 2021; Xu et al., 2024). At present, there is limited understanding of the functional role of *OsRDH* in rice developmental regulation or its possible contribution to the formation of rice quality characteristics.

In previous studies, we identified *OsAAP6* as a major quantitative trait locus (QTL) gene that positively regulates grain protein accumulation and nutritional quality in rice (Peng et al., 2014a, Peng et al., 2018). Subsequent investigations into the molecular basis of *OsAAP6*-mediated control of grain protein content revealed that *OsGAPC3* physically interacts with *OsAAP6* and modulates its transcriptional activity. Through this regulatory relationship, *OsGAPC3* influences the accumulation of major storage compounds in rice grains and thereby affects multiple quality-related traits (Peng et al., 2024b). Transcriptome sequencing further demonstrated that *OsRDH* expression is significantly induced in the *Osgapc3* mutant, whereas it is markedly suppressed in *OsGAPC3* overexpression lines (Peng et al., 2024b). Additional analyses indicated that *OsRDH* is constitutively expressed in rice, and its encoded protein is targeted to chloroplasts. Functional disruption of *OsRDH* led to pronounced alterations in grain nutrient composition, which in turn exerted substantial effects on rice appearance, processing performance, and nutritional quality. Moreover, *OsRDH* was found to participate in the regulation of genes associated with gibberellin (GA) and ABA metabolic pathways, thereby influencing seed germination and primary root growth in rice. Collectively, these results demonstrate that *OsRDH* plays a broad regulatory role in rice quality formation and developmental processes, including early seedling growth. This work provides important insights and a potential genetic target for the molecular breeding of rice varieties with improved quality traits.

## 2 Materials and methods

### 2.1 Generation of rice *Osrdh* mutants

To explore the biological function of *OsRDH* in rice, we generated an *OsRDH* gene-edited line using the wild-type Zhonghua 11 (ZH11) as the genetic background. Specific target sequences within *OsRDH* were selected, and CRISPR/Cas9-mediated genome editing was applied to direct the sgRNA-Cas9 complex to a protospacer adjacent motif (PAM) site in the *OsRDH* locus. An expression vector for gene editing was constructed and ligated into the final gene editing vector pYLCRISPR/Cas9-MH (B), resulting in the generation of a CRISPR/Cas9-based mutant targeting *OsRDH* knockout. The genetic transformation of the rice *Osrdh* mutant was carried out by Wuhan Tianwen Biotechnology Company Limited. The presence of mutations in the *OsRDH* gene was assessed through PCR amplification followed by Sanger sequencing, verifying that the gene was effectively disrupted and that no unintended off-target effects occurred (Peng et al., 2024b). Through sequencing and segregation analysis of the T_1_ generation, we isolated the *Osrdh* mutant using PCR and Sanger sequencing techniques.

### 2.2 Construction of subcellular localization vectors

The full-length coding sequence (CDS) of *OsRDH* (LOC_Os03g02460) was retrieved from the National Rice Database (https://www.ricedata.cn/gene/) for the construction of the subcellular localization vector. Primers were designed using the NCBI website to amplify the target fragment from rice ZH11. The cDNA fragment of *OsRDH* was initially inserted into the intermediate vector PMD-18T, resulting in the recombinant plasmid PMD-18T-*OsRDH*. The integrity and correctness of the inserted *OsRDH* sequence were confirmed by sequencing. Following sequence verification, the target fragment was excised from PMD-18T-*OsRDH* using the restriction enzymes *Kpn* I and *Xba* I and subsequently ligated into the pM999–616 vector (carrying *green fluorescent protein gene*, *GFP*). This procedure yielded the subcellular localization construct pM999-616-*OsRDH* for downstream analysis of *OsRDH* localization.

### 2.3 RNA extraction and quantitative real-time polymerase chain reaction

Total RNA was extracted using TRIzol reagent according to the manufacturer’s instructions (TAKARA). RNA samples were reverse-transcribed into cDNA using the R202–02 HyperScript III RT SuperMix for qPCR with gDNA Remover. qRT-PCR analyses were carried out on an ABI 7300 system with 2× SYBR Green Fast qPCR Master Mix (YiFeiXue Bio-Technology, China). The amplification protocol included an initial denaturation at 95 °C for 30 seconds, followed by 40 cycles consisting of 15-second denaturation at 95 °C and 30-second combined annealing/extension at 60 °C. β-Actin was used as the reference gene for normalization, and relative transcript levels were determined using the 2*^−ΔΔCT^* method (Peng et al., 2024c). Each reaction was conducted in triplicate to provide biological replicates.

### 2.4 Analysis of expression patterns

Primers for *OsRDH* were designed using its cDNA sequence and assessed through the NCBI website. RNA was extracted from different tissues of ZH11 rice, including young roots, stems, and leaves, as well as roots, stems, leaves, and panicles at the heading stage. The RNA was converted into cDNA, and tissue-specific expression of *OsRDH* was determined by qRT-PCR with β-Actin as the internal control. Each experiment was performed in triplicate for biological replication.

### 2.5 Subcellular localization

Protoplasts were isolated from 12-day-old rice seedlings, and the subcellular localization vector pM999-616-*OsRDH* was individually introduced into protoplasts derived from rice leaf sheath cells for expression. As a control, the PM999-GFP empty vector was also introduced into rice protoplasts. Fluorescence signals in the protoplasts were then observed using a laser confocal microscope (Leica TCS SP8) (Peng et al., 2023).

### 2.6 Rice growth status and trait measurement

Rice was cultivated at the Xinyang Normal University experimental station, with plants spaced 16.5 cm apart within rows and 26 cm between rows. Field management adhered to local agronomic practices. Harvested seeds were air-dried naturally and stored at room temperature for a minimum of three months prior to analysis. Only fully filled grains were used for the evaluation of rice quality and yield-related traits. Measurements of protein, amylose, free fatty acids, and taste value were conducted using a near-infrared grain analyzer (INFRAEC Nova) (Peng et al., 2023). Gelatinization temperature was assessed using the alkali spreading method, and gel consistency of polished grains was determined according to the national standard (GB/T17891-1999). All assays were carried out in triplicate.

### 2.7 Transmission electron microscopy analysis

For ultrastructural analysis, transverse sections of 10 DAF rice seeds were fixed in buffered 2.5% glutaraldehyde for 12 hours and subsequently processed via cryosectioning. Protein bodies in the endosperm were observed under a Tecnai G2 F20 transmission electron microscope, and their areas were quantified using ImageJ software.

### 2.8 Scanning electron microscopy observation

The middle of the rice grain was struck with the back of a blade to induce natural fracture, and the fractured section was then sliced with a knife to prepare samples approximately 2–3 mm thick (Peng et al., 2024a). One subset of the grains was examined under an optical microscope, and the cross-sections of a separate subset were visualized using a cold field emission SEM (Regulus 8220).

### 2.9 Measurement of chlorophyll content and SPAD value in plants

Chlorophyll content was determined by measuring the absorbance of chlorophyll extract at a specific wavelength using a spectrophotometer, based on the absorption of visible light by the chlorophyll extract. Each sample was measured three times and subjected to *t*-test for statistical analysis between groups. SPAD values were measured using a SPAD instrument on leaves at the heading stage, with measurements taken from the same part of the leaves at the same stage for both *Osrdh* and WT plants (Hu et al., 2021). The levels of IAA, ABA, and GA_3_ hormones in *Osrdh* leaves at the heading stage were analyzed by Guangzhou Haojing Biotechnology Company Limited.

### 2.10 Rice seed germination experiment

Rice seeds were disinfected with 0.1% mercuric chloride for 45 minutes, then rinsed three times with tap water, followed by distilled water, and placed in a 25 °C incubator for one day to accelerate germination. Clean 12*12cm Petri dishes were numbered for the experiment. Normal germination tests and treatments with 5 μM hormones IAA, ABA, and GA3 were performed separately (Huang et al., 2024). Filter paper, cut to correspond to the size of the base of each Petri dish, was positioned inside each dish. The germinated rice seeds were carefully arranged in the Petri dish using tweezers, with the shoot tips facing upward and the embryo parts aligned on the same side. After covering the dish with its lid, it was placed in the dark at room temperature for seven days. The germination status of the seeds was recorded daily, and the growth of the rice seedlings was observed and photographed. Three replicate experiments were conducted.

### 2.11 Rice root development experiment

After peeling and disinfecting the rice seeds, place them in a 50 mL centrifuge tube. Disinfect the seeds with 75% ethanol for 2–3 minutes, then discard the alcohol. Soak the seeds in 0.15% mercuric chloride for 15 minutes, shaking the centrifuge tube gently several times during the process. After soaking, discard the mercuric chloride and rinse the seeds 10–12 times with sterile distilled water on a clean bench to remove as much mercuric chloride as possible from the seed surface. The treated seeds were placed in half-strength MS medium and incubated in a growth chamber at 28 °C under constant light for seven days. Seedling growth was subsequently monitored and recorded (Peng et al., 2023).

### 2.12 Proteomics analysis

Endosperm of mature seeds from the *Osrdh* mutant and wild-type plants were analyzed using iTRAQ-based proteomics. To evaluate the quality and reproducibility of the acquired data, principal component analysis (PCA) and relative standard deviation (RSD) distributions were assessed. Proteins exhibiting differential expression were identified based on fold-change thresholds and associated P values derived from relative quantification. Subsequently, the biological functions and metabolic pathways associated with these differentially expressed proteins (DEPs) were examined through Gene Ontology (GO) and Kyoto Encyclopedia of Genes and Genomes (KEGG) enrichment analyses (Peng et al., 2024b).

### 2.13 Plant hormone detection and analysis

The extraction and quantitative determination of abscisic acid (ABA), gibberellin A₃ (GA₃), and indole-3-acetic acid (IAA) were performed using liquid chromatography-tandem mass spectrometry (LC-MS/MS). A 100 mg sample of rice leaves at the heading stage was ground into fine powder in liquid nitrogen, and 4 mL of extraction solution (isopropanol: glacial acetic acid = 99:1, v/v) containing 20 ng of deuterium-labeled internal standards ([^2^H_6_]-ABA, [^2^H_2_]-GA_3_, and [^2^H_5_]-IAA) was added. Detection was carried out in multiple reaction monitoring mode, with negative ion mode for ABA and IAA, and positive ion mode for GA_3_ (Hu et al., 2021). Absolute quantification was conducted using the isotope internal standard method, and each sample was set with three biological replicates.

### 2.14 Haplotype analysis

Single-nucleotide polymorphisms (SNPs) in the coding region of *OsRDH* were analyzed. Sequence data from 533 cultivated rice accessions were obtained from the Rice Genome Project database (https://ricevarmap.ncpgr.cn/vars_genotype/) in 2023. Representative haplotypes were identified based on polymorphic sites within the coding sequence, and their distribution and frequencies among different rice populations were subsequently evaluated.

## 3 Results

### 3.1 *OsRDH* is constitutively expressed, and the encoded protein is localized to chloroplasts

**Figure 1:**
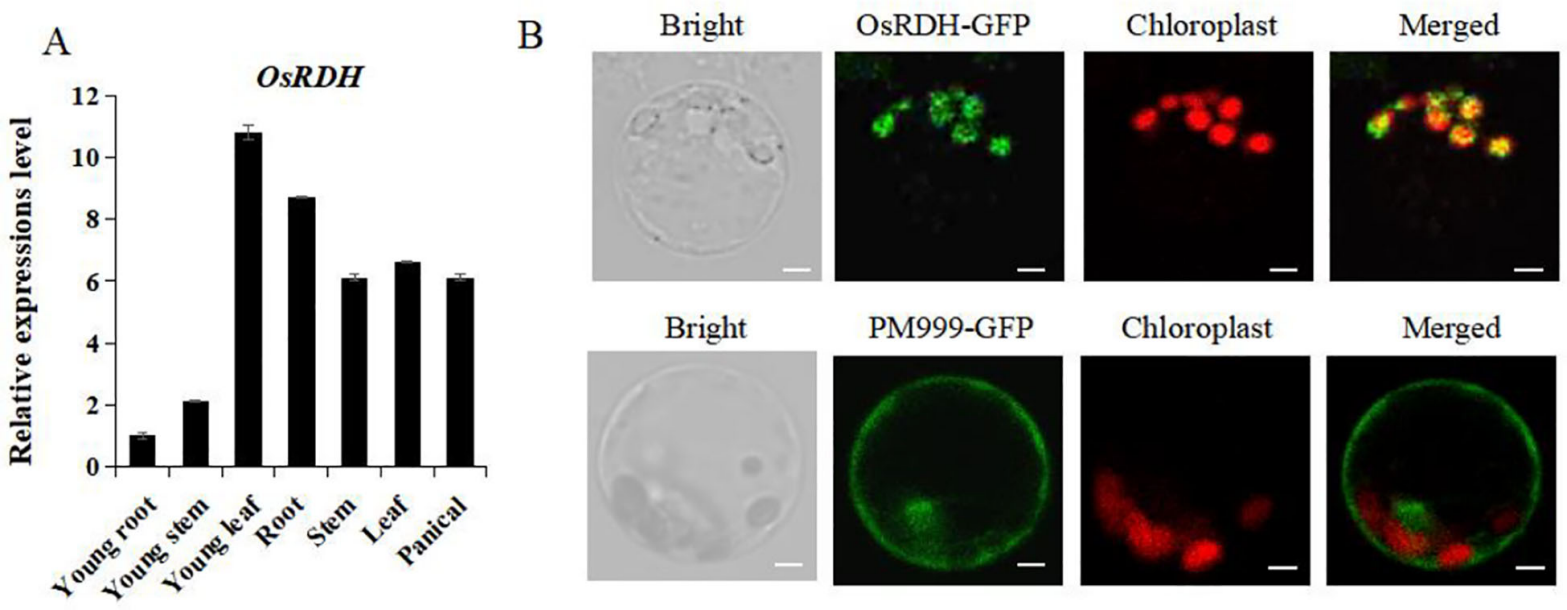
Expression pattern analysis and subcellular localization of rice *OsRDH*. **(A)** Analysis of *OsRDH* transcript levels across multiple rice tissues. Each experiment was performed in triplicate for biological replication. **(B)** GFP subcellular localization, PM999-GFP is an empty vector (serving as a control). Error bars represent the standard error of the mean (SEM). Subcellular localization of the *OsRDH* protein and empty vector PM999-GFP. Green, red, and yellow fluorescence represent GFP, chloroplast autofluorescence, and merged fluorescence, respectively. Scale bar: 5 μm.

To investigate the tissue-specific expression of *OsRDH*, total RNA was isolated from seven rice tissues, including young roots, young stems, young leaves, roots at the heading stage, stems at the heading stage, flag leaves, and young panicles. Quantitative real-time PCR (qRT-PCR) analysis showed that *OsRDH* transcripts were detectable in all examined tissues, consistent with the expression pattern of *OsRDH* (LOC_Os03g02460) reported in the CREP database (**Supplementary Figure 1**), indicating that the gene is constitutively expressed. Among the tissues tested, expression was relatively higher in young leaves and lower in young roots and stems (**Figure 1A**).

For subcellular localization, the *OsRDH* coding sequence was fused in-frame to a green fluorescent protein (GFP) reporter (*OsRDH*-GFP) and transiently expressed in rice protoplasts, with an empty GFP vector (PM999-GFP) serving as a control. Confocal laser scanning microscopy revealed that fluorescence from PM999-GFP was distributed throughout the cell, including the plasma membrane, cytoplasm, and nucleus. In contrast, *OsRDH*-GFP fluorescence was exclusively observed in chloroplasts, demonstrating that *OsRDH* is localized to chloroplasts (**Figure 1B**).

### 3.2 Generation of rice *Osrdh* mutant

**Figure 2:**
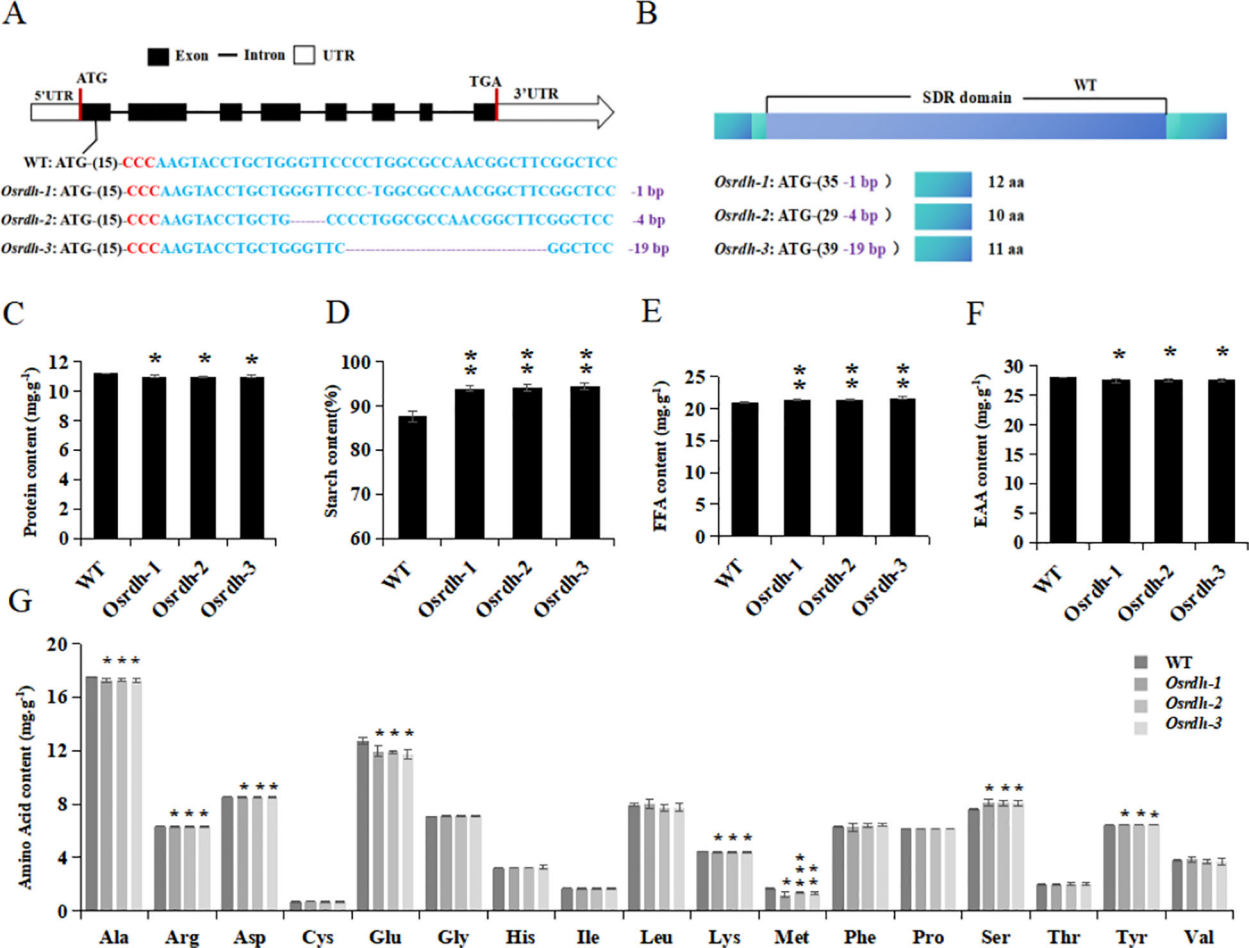
Creation of the rice *Osrdh* mutant and detection and analysis of main storage substances in its grains. **(A)** Identification of *Osrdh* mutation type. **(B)** Schematic diagram of amino acid changes caused by *Osrdh* mutation. **(C)** Protein content in grains of *Osrdh* mutant. **(D)** Total starch content in grains of *Osrdh* mutant. **(E)** Free fatty acid content in grains of *Osrdh* mutant. **(F)** Essential amino acid content in grains of *Osrdh* mutant. **(G)** Amino acid content in grains of *Osrdh* mutant. Note: WT, Wild type. Each experiment was performed in triplicate for biological replication. Statistical significance was determined using a two-tailed *t-*test: ***P < 0.001, **P < 0.01, *P < 0.05. Error bars represent the standard error of the mean (SEM).

An *Osrdh* mutant was developed to study the function of *OsRDH* in rice, using the Zhonghua 11 (ZH11) cultivar as the wild-type background. A single guide RNA (sgRNA) was constructed to target a PAM site located in the first exon of the *OsRDH* gene. The CRISPR/Cas9 gene-editing system was employed to induce site-specific cleavage at the target locus. The sgRNA was cloned into the pYLCRISPR/Cas9-MH (B) expression vector, and the construct was introduced into rice via *Agrobacterium tumefaciens*–mediated transformation. A total of 48 independent transgenic lines were obtained, among which 42 lines carried mutations in *OsRDH* (**Supplementary Figures 2A, B**). Sequence analysis identified three distinct mutation types in the *Osrdh* mutants: *Osrdh*-1 (-1 bp), *Osrdh*-2 (-4 bp), and *Osrdh*-3 (-19 bp) (**Figure 2A**, **Supplementary Figure 2C**). Deletions in *Osrdh*-1 and *Osrdh*-3 resulted in frameshift mutations within the first exon, whereas the deletion in *Osrdh*-2 caused a premature stop codon, leading to truncated protein products (**Figure 2B**).

### 3.3 *OsRDH* influences the accumulation of key storage compounds in rice grain

**Figure 3:**
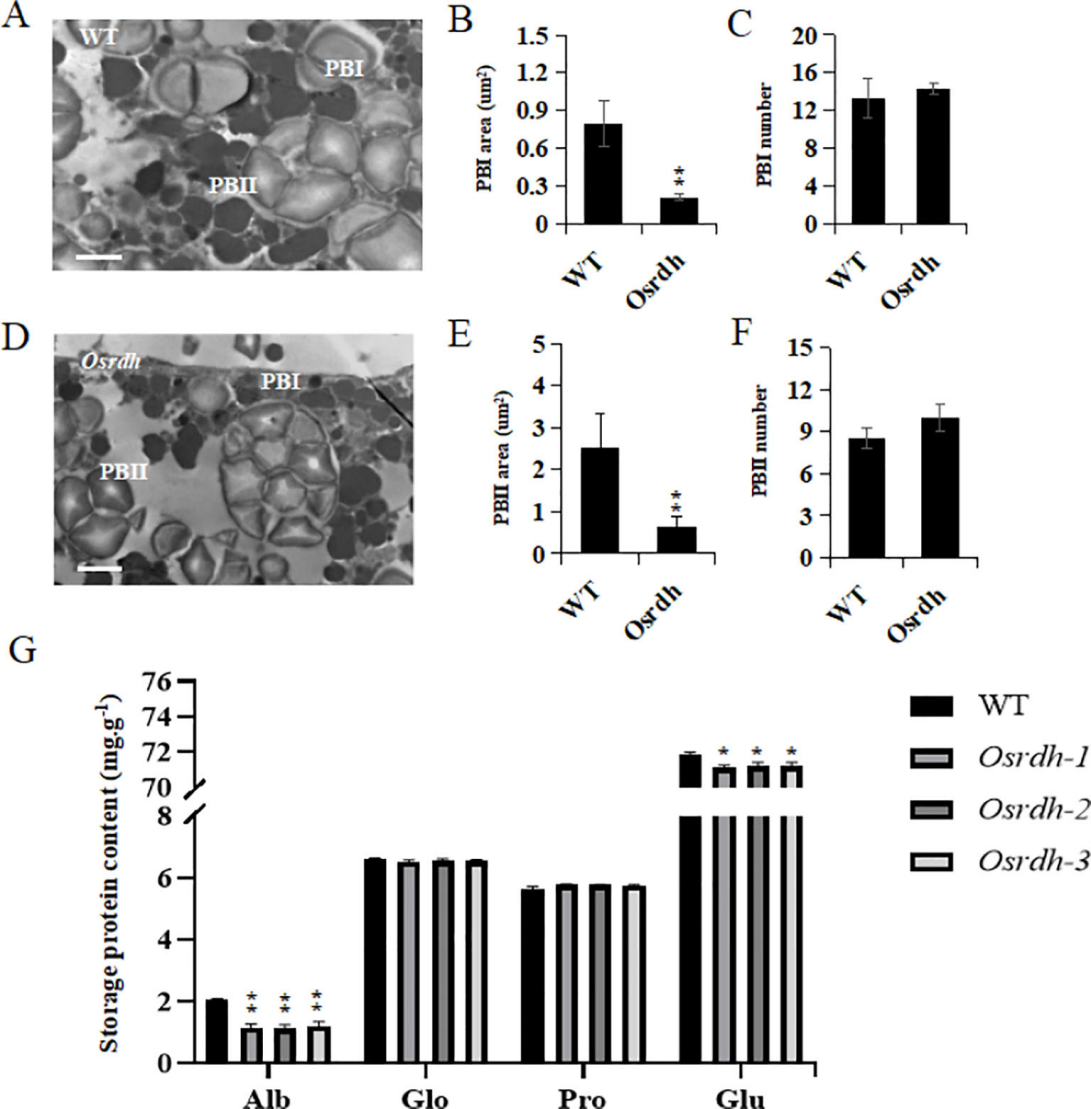
Assessment of protein body development in the endosperm of *Osrdh* mutants by transmission electron microscopy. **(A)** Observation of protein body ultrastructure in wild-type rice endosperm using transmission electron microscopy. **(B)** Observation of endosperm protein body ultrastructure in *Osrdh* rice mutants by transmission electron microscopy, scale bar: 5 μm. **(C)** Area of endosperm protein body I in the *Osrdh* mutant. **(D)** Number of endosperm protein bodies I in the *Osrdh* mutant. **(E)** Area of endosperm protein body II in the *Osrdh* mutant. **(F)** Number of endosperm protein bodies II in the *Osrdh* mutant. **(G)** Seed storage protein content in the *Osrdh* mutant, including Albumin, globulin, prolamine, and glutelin. Note: WT, Wild type. Each experiment was performed in triplicate for biological replication. A two-tailed *t*-test was applied to assess statistical differences, with **P < 0.01 and *P < 0.05 representing levels of significance. Error bars correspond to the standard error of the mean (SEM).

Grain protein, starch, and free fatty acid (FFA) contents were analyzed to examine the influence of *OsRDH*. In *Osrdh* mutants, grain protein (reduced by 2.0%) levels were significantly lower, while both starch (increased by 2.3%) and FFA (increased by 2.9%) levels were significantly higher relative to wild-type grains (**Figures 2C–E**). Amino acid profiling further revealed that the levels of alanine, arginine, aspartic acid, glutamic acid, lysine, and methionine, as well as the total essential amino acid content (reduced by 1.8%), were significantly decreased in *Osrdh* mutants. In contrast, the contents of serine and tyrosine were significantly elevated (**Figures 2F, G**). Endosperm samples collected at 10 days after flowering (DAF) were examined by TEM to determine whether *OsRDH* influences the formation and development of protein bodies. Quantitative analysis showed that the cross-sectional areas of both Protein Body I (PB I) and Protein Body II (PB II) were significantly reduced in *Osrdh* mutants, whereas the numbers of PB I and PB II did not differ significantly from those of the wild type (**Figures 3A–F**). Subsequent analysis of the four major classes of storage proteins revealed that the contents of albumin and glutelin were significantly decreased in *Osrdh* mutants, while globulin and prolamin levels remained unchanged (**Figure 3G**). Collectively, these results indicate that the reduced sizes of PB II and PB II and the decreased accumulation of albumin and glutelin account for the lower protein content observed in *Osrdh* mutants grains. Overall, mutation of *OsRDH* significantly alters the accumulation of major storage compounds, including protein, starch, essential amino acids, and free fatty acids, in rice grains.

### 3.4 Proteomic analysis of *Osrdh* mutant endosperm

**Figure 4:**
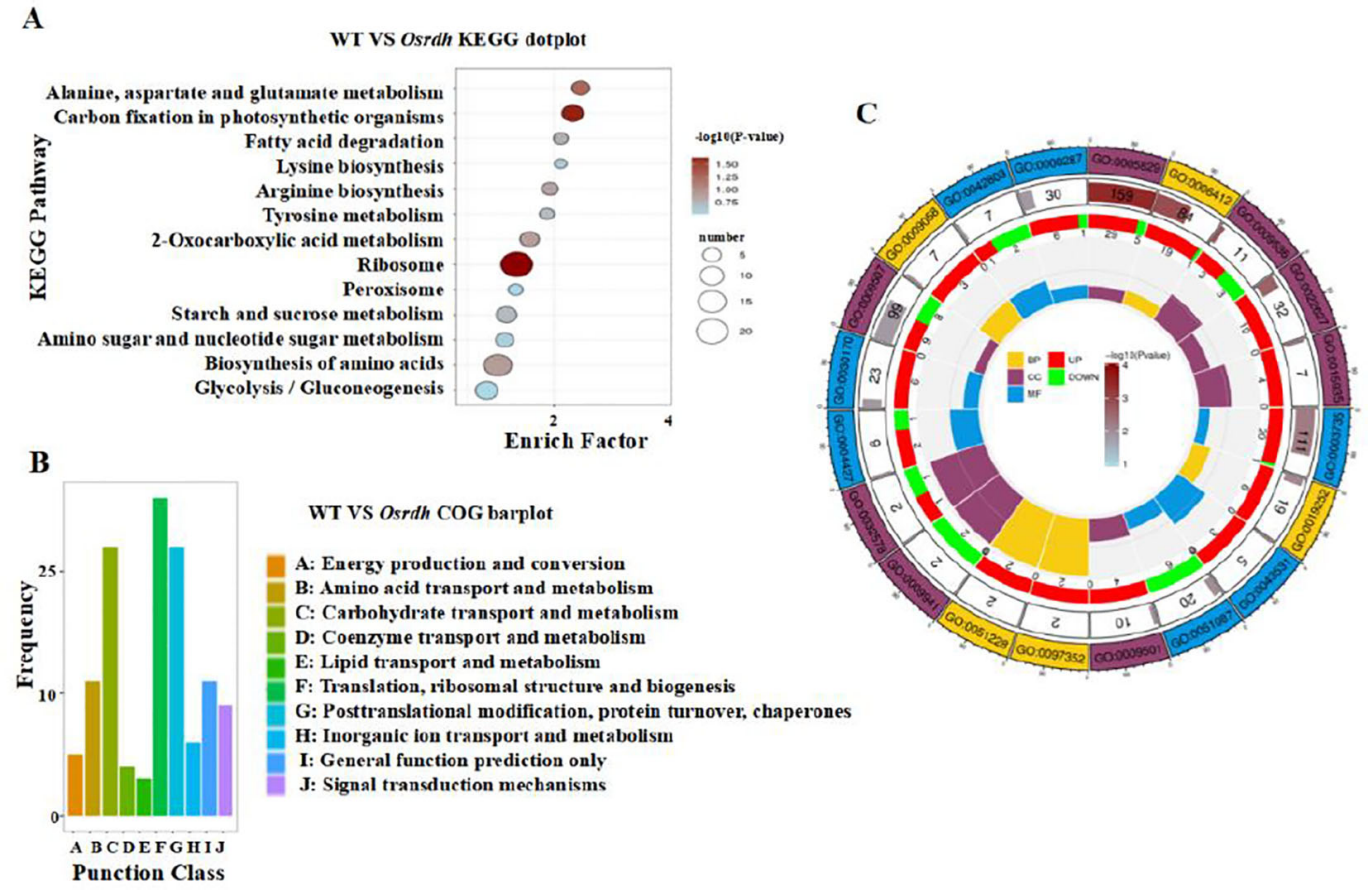
Proteomic profiling of endosperm from *Osrdh* mutants. **(A)** KEGG pathway enrichment of up- and downregulated DEPs is illustrated, with each circle representing a pathway. The vertical axis lists the pathway names, and the horizontal axis reflects the enrichment factor for each pathway. **(B)** Functional categorization of DEPs according to COG is illustrated, with COG clusters along the horizontal axis and the number of annotated DEPs along the vertical axis. **(C)** GO enrichment analysis of DEPs is presented as a multi-layered circle chart: the first layer denotes the total number of GO terms, the second layer indicates the number of proteins enriched in each term, the third layer shows the statistical significance of DEP enrichment (darker colors correspond to higher significance), and the fourth layer represents the proportion of DEPs in each GO term compared with all identified proteins.

In light of the significant reduction in protein levels in *Osrdh* mutant grains, endosperm of mature seeds was subjected to proteomic analysis to examine changes in the abundance of storage-related proteins. The relative standard deviation (RSD) of biological replicates indicated high data stability and low intra-group variability, demonstrating good reproducibility of the proteomic dataset. Proteins exhibiting differential expression were identified using fold-change and *P*-value criteria. Those with FC ≥ 1.20 and *P* ≤ 0.05 were classified as upregulated, while proteins with FC ≤ 0.83 (1/1.20) and *P* ≤ 0.05 were defined as downregulated (**Supplementary Figure 3A**). In total, 189 DEPs were detected in the *Osrdh* mutant endosperm, including 132 upregulated and 57 downregulated proteins (**Supplementary Figure 3B**, **Supplementary Table 1**). Predicted subcellular localization indicated that the majority of DEPs were present in the nucleus, cytoplasm, plasma membrane, and mitochondria (**Supplementary Figure 3C**). Functional classification using the Clusters of Orthologous Groups (COG) database showed that the DEPs were mainly associated with amino acid transport and metabolism, carbohydrate transport and metabolism, and related biological processes (**Supplementary Figure 3D**). To further characterize the biological pathways associated with the DEPs, GO and KEGG annotations and enrichments were performed. KEGG pathway analysis showed that downregulated DEPs in the *Osrdh* mutant were mainly associated with pathways including amino acid biosynthesis, starch and sucrose metabolism. Upregulated DEPs, on the other hand, were predominantly linked to ribosome biogenesis, carbon fixation in photosynthetic organisms, and α-linolenic acid metabolism (**Figures 4A–C**). Based on functional annotation of rice genes (https://www.ricedata.cn/gene/), several genes corresponding to the 189 DEPs, including *OsBTF3, OsPIMT2, OsHSP26.7, OsDHAR1, OsPDIL2, OsPHY2, OsAlba1, OsPUL3, FLO12, OsGluA2, OsDHDPS, gpa2, OsCDC48, OsPK1, OsGAPC3, OsBT1, OsAGPL2*, and *SBDCP1*, were identified in involving in protein and starch metabolism as well as seed development processes and plant growth (**Supplementary Table 2**). Interestingly, OsPUL3, FLO12, OsGluA2, Ospdc2, OsGAPC3, OsBT1, OsAGPL2, and SBDCP1 were involved in starch biosynthesis and accumulation processes, and they were significantly upregulated in the endosperm of *Osrdh* mutants (**Supplementary Table 2**), which is consistent with the phenotype of significantly increased starch content in *Osrdh* mutant grains (**Figure 2D**). Meanwhile, proteins closely related to grain protein synthesis and accumulation, as well as rice growth and development, such as OsPIMT2, OsPDIL2, OsPHY2, OsHSP26.7, OsBTF3, and OsAlba1, were significantly downregulated in the endosperm of *Osrdh* mutants (**Supplementary Table 2**). This is consistent with the phenotype of *Osrdh* mutants, such as decreased protein content in grains and reduced rice plant growth. Taken together, the data indicate that *OsRDH* plays a pivotal role in controlling the biosynthesis and deposition of major storage substances, especially starch and protein, within the rice endosperm.

### 3.5 *OsRDH* affects the expression of genes involved in major storage compound metabolism in rice grains

**Figure 5:**
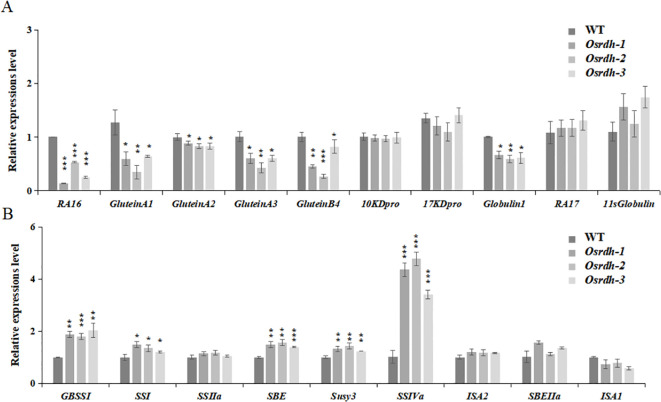
Evaluation of transcriptional levels of starch and protein metabolism genes in *Osrdh* mutant grains during grain filling. **(A)** Quantification of expression for protein metabolism-associated genes in *Osrdh* mutant grains. **(B)** Quantification of expression for starch metabolism-associated genes in *Osrdh* mutant grains. WT, Wild type. Each experiment was performed in triplicate for biological replication. A two-tailed t-test was used to determine statistical differences, where ***P < 0.001, **P < 0.01, and *P < 0.05 indicate levels of significance. Error bars correspond to the standard error of the mean (SEM).

Given the pronounced effects of *OsRDH* mutation on starch and protein accumulation, the expression of 19 genes involved in protein and starch metabolism was analyzed in *Osrdh* mutant grains during the grain-filling stage (15 DAF). Transcripts of protein synthesis and metabolism genes, including *RA16, GlutelinA1, GlutelinA2, GlutelinA3, GlutelinB4*, and *Globulin1*, were significantly reduced in the *Osrdh* mutant (**Figure 5A**; **Supplementary Table 3**). In contrast, starch biosynthesis-related genes, such as *GBSSI, SSI, SSIIa, SSIVa, SBE* (including *SBEIIa*), *ISA*, and *Susy3*, exhibited significantly increased expression levels (**Figure 5B**; **Supplementary Table 4**). These transcriptional alterations align with the phenotypic changes observed in the *Osrdh* grains, which display decreased protein content and enhanced starch accumulation.

### 3.6 The *OsRDH* mutation improves appearance, processing, and cooking and eating quality in rice

**Figure 6:**
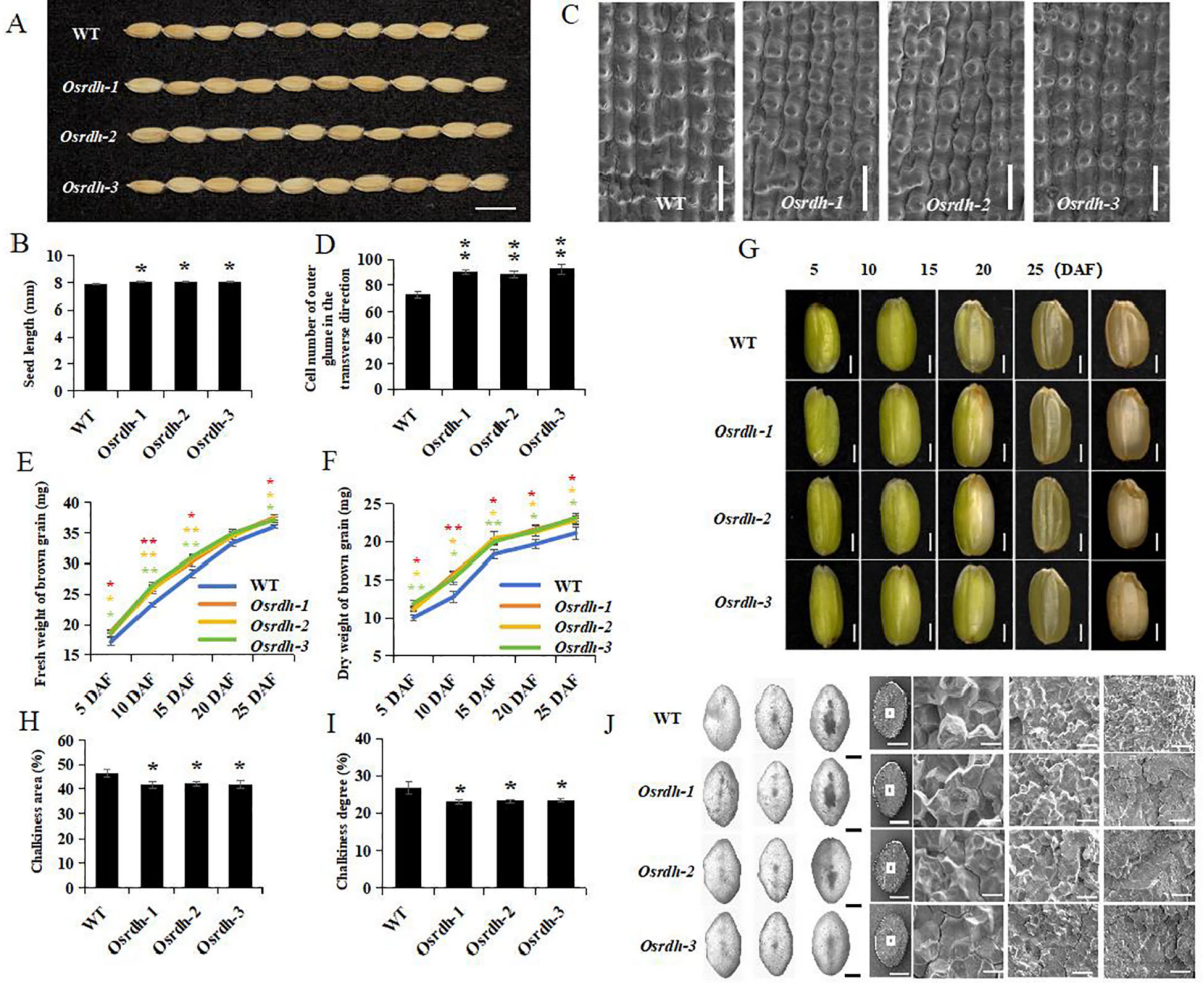
Detection and analysis of appearance quality traits of *Osrdh* mutant. **(A)** Seed grain type of *Osrdh* mutant, scale: 1 cm. **(B)** Statistical analysis of seed grain length of *Osrdh* mutant. **(C)** Phenotype of mature seed chaff of *Osrdh* mutant, scale: 200 μm. **(D)** Statistical analysis of the number of longitudinal cells in chaff cells of *Osrdh* mutant. **(E)** Dynamic change analysis of fresh weight during the grain filling stage of *Osrdh* mutant. **(F)** Dynamic change analysis of dry weight during the grain filling stage of *Osrdh* mutant. **(G)** Dehulled seed phenotype of *Osrdh* mutant during the grain filling stage on days 5, 10, 15, 20, and 25 after flowering (DAP), scale: 1 mm. **(H)** Statistical analysis of chalkiness area of *Osrdh* mutant rice. **(I)** Statistical analysis of chalkiness degree of *Osrdh* mutant rice. **(J)** Optical microscopy observation of chalkiness areas of *Osrdh* mutant rice and scanning electron microscopy observation of rice starch granules, scale: 1 mm; Scales: From left to right, scales are: 1 mm, 10 μm, 20 μm, and 50 μm. Note: WT, Wild type. Each experiment was performed in triplicate for biological replication. Significant differences were based on a two-tailed *t*-test, **P<0.01, *P < 0.05. Error bars, Standard error of the mean (SEM).

To evaluate the effects of *OsRDH* on rice quality traits, the grain morphology of *Osrdh* mutant seeds was first examined. The results showed that the grain length was significantly increased in *Osrdh* mutant, whereas grain width and thickness did not differ significantly from the wild type (**Figures 6A, B**; **Supplementary Figures 4A–C**). To elucidate the basis for the increased grain length, seed glumes were observed using scanning electron microscopy (SEM) (**Figure 6C**). Quantitative analysis revealed that the number of outer glume cells was significantly higher in *Osrdh* mutants, while the transverse cell length remained unchanged (**Figure 6D**; **Supplementary Figure 3D**), suggesting that the enhanced grain length primarily resulted from increased cell proliferation rather than cell expansion. Grain development was further assessed by measuring dehulled fresh and dry seed weights at 5, 10, 15, 20, and 25 days after pollination (DAP) during the grain-filling stage. The *Osrdh* mutants consistently exhibited higher fresh and dry weights and longer grains than the wild type (**Figures 6E–G**).

Chalkiness traits were subsequently evaluated. Both the chalkiness area and chalkiness degree were significantly reduced in *Osrdh* mutant grains (**Figures 6H, I**), whereas the chalkiness rate did not differ significantly (**Supplementary Figure 4E**). Light microscopy showed that the reduction in chalkiness was mainly attributable to a decreased chalky core region. SEM analysis further revealed that the starch granules in the *Osrdh* mutant were more densely packed, displaying a uniform polygonal morphology and a more compact arrangement (**Figure 6J**). These structural features are consistent with improved appearance quality.

Assessment of processing, cooking, and eating quality revealed that *Osrdh* mutant rice exhibited a marked reduction in amylose content, while both gel consistency and gelatinization temperature remained comparable to the wild type (**Supplementary Figures 5A–C**). Moreover, the head rice percentage was significantly increased, while brown rice and milled rice percentages were not significantly affected (**Supplementary Figures 5D–F**). Together, these results indicate that the *OsRDH* mutation enhances processing quality by increasing head rice yield and improves cooking and eating quality by reducing amylose content.

### 3.7 Chlorophyll content decreases in *Osrdh* mutant

**Figure 7:**
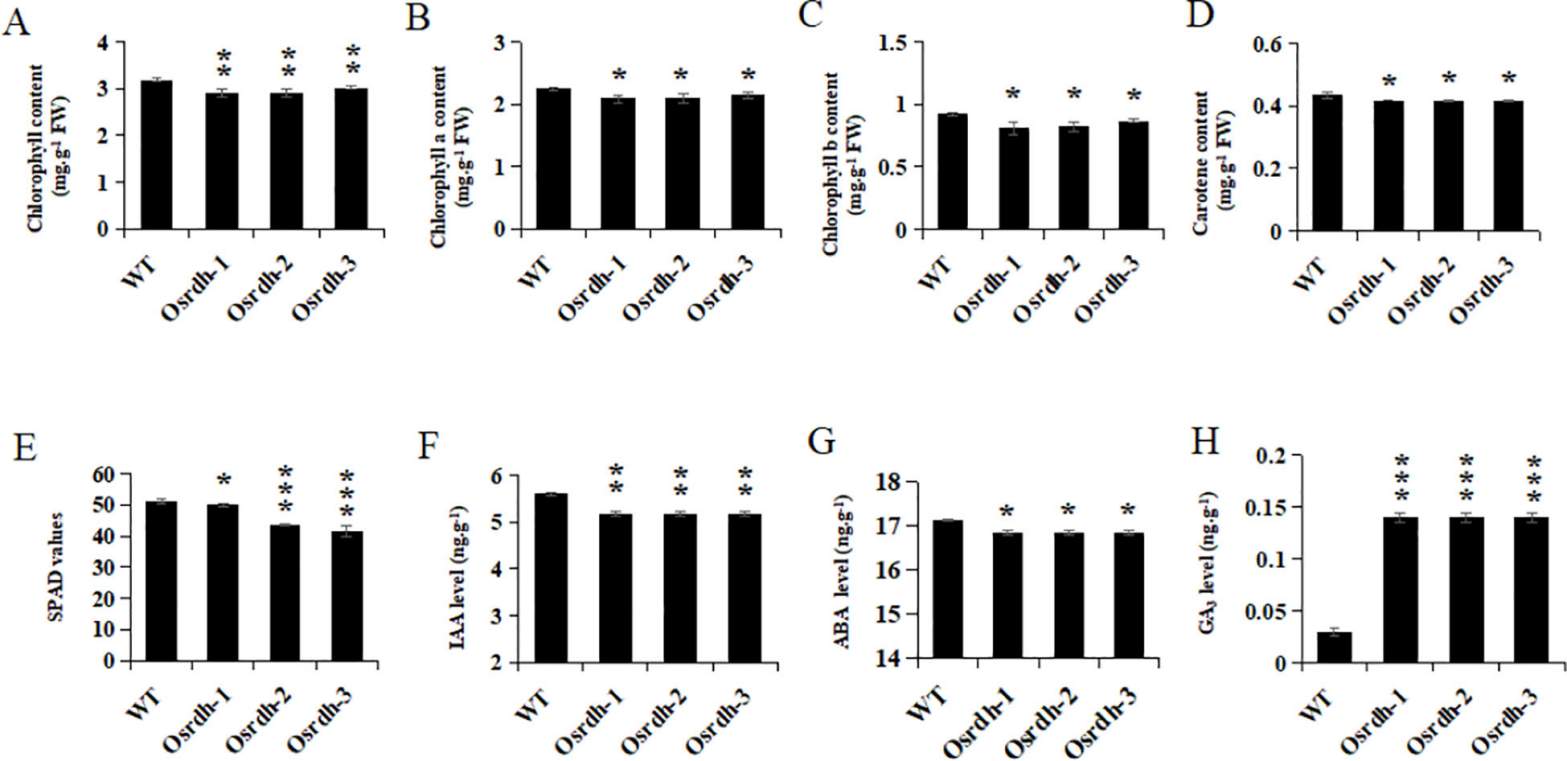
Detection and analysis of chlorophyll content and common plant hormone levels in flag leaves of *Osrdh* mutant. **(A)** Quantitative analysis of total chlorophyll content in flag leaves of *Osrdh* mutants at the heading stage. **(B)** Quantification of chlorophyll a levels in flag leaves of *Osrdh* mutants at the heading stage. **(C)** Quantification of chlorophyll b levels in flag leaves of *Osrdh* mutants at the heading stage. **(D)** Measurement of carotenoid content in flag leaves of *Osrdh* mutants at the heading stage. **(E)** SPAD value of flag leaves of *Osrdh* mutant at heading stage. **(F)** Detection and analysis of IAA level in flag leaves of *Osrdh* mutant at heading stage. **(G)** Detection and analysis of ABA level in flag leaves of *Osrdh* mutant at heading stage. **(H)** Detection of GA_3_ level in flag leaves of *Osrdh* mutant at heading stage. Note: WT, Wild type. Each experiment was performed in triplicate for biological replication. Statistical significance was evaluated using a two-tailed *t*-test (***P < 0.001, **P < 0.01, *P < 0.05). Error bars indicate the standard error of the mean (SEM).

Given the chloroplast localization of the *OsRDH* protein and its potential role in photosynthesis, chlorophyll content was measured in flag leaves of the *Osrdh* mutant at the heading stage. Compared with the wild type, flag leaves of the *Osrdh* mutant exhibited significantly lower levels of total chlorophyll, chlorophyll a, chlorophyll b, carotenoids, as well as reduced SPAD values (**Figures 7A–E**). These reductions suggest that the *OsRDH* mutation impairs photosynthetic capacity in flag leaves, which may consequently affect plant growth and development. Because *OsRDH* expression has been reported to be inhibited by ABA (Dickinson et al., 2021), endogenous levels of IAA, ABA, and GA_3_ were quantified in flag leaves at the heading stage. The results showed that IAA and ABA were significantly decreased, whereas GA_3_ levels were significantly increased in *Osrdh* mutants relative to the wild type (**Figures 7F–H**). Collectively, these findings suggest that altered phytohormone homeostasis in the *Osrdh* mutant may contribute to the observed reduction in chlorophyll content.

### 3.8 *OsRDH* mutation is detrimental to rice yield

**Figure 8:**
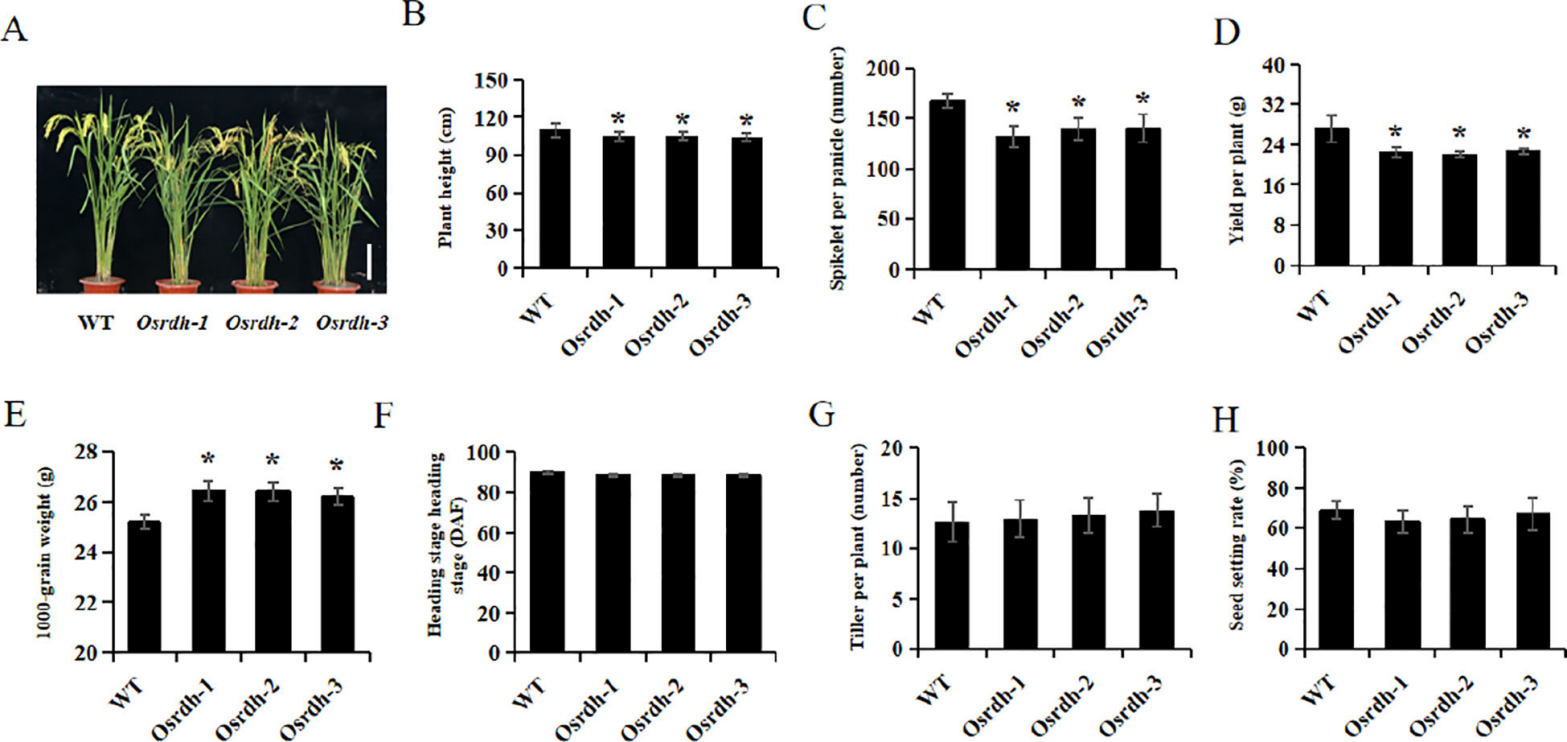
Yield-related trait characterization of *Osrdh* mutant rice **(A)** Statistical analysis of plant height in *Osrdh* mutant. **(B)** Phenotype observation of *Osrdh* mutant plants, scale: 30 cm. **(C)** Statistical analysis of the number of spikelets per panicle in *Osrdh* mutant. **(D)** Statistical analysis of the yield per plant in *Osrdh* mutant. **(E)** Statistical analysis of 1000-grain weight in *Osrdh* mutant. **(F)** Statistical analysis of heading date in *Osrdh* mutant. **(G)** Statistical analysis of tillering in *Osrdh* mutant. **(H)** Statistical analysis of seed setting rate in *Osrdh* mutant. WT, Wild type. Each experiment was performed in triplicate for biological replication. Significant differences were based on a two-tailed *t*-test, *P < 0.05. Error bars, Standard error of the mean (SEM).

Field-based assessments were performed to investigate the influence of *OsRDH* disruption on agronomic traits associated with yield. The *Osrdh* mutant exhibited a significant decrease in plant height (reduced by 4.8%), spikelet number per panicle (reduced by 18.5%), and yield per plant (reduced by 17.6%) when compared with the wild-type, while the 1000-grain weight was significantly increased by 4.7% (**Figures 8A–E**). No significant changes were detected in tillering ability, heading time, or seed-setting rate (**Figures 8F–H**). Collectively, these results demonstrate that *OsRDH* deficiency leads to a reduction in rice yield.

### 3.9 Analysis of *OsRDH* haplotypes

**Figure 9:**
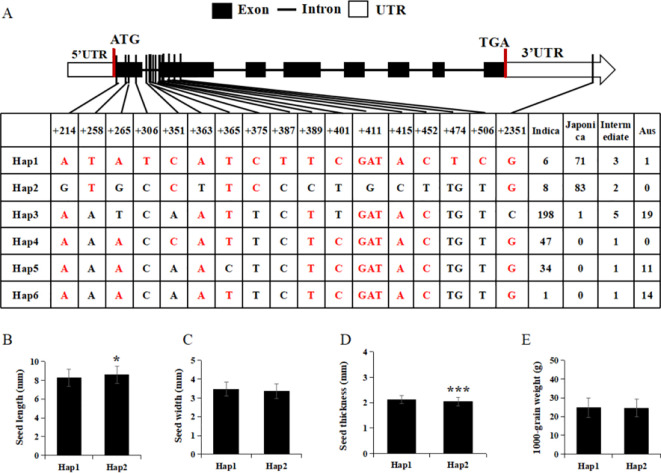
Haplotype analysis of *OsRDH*-related grain shape traits. **(A)** Haplotype identification in the coding region of *OsRDH*. **(B)** Comparison of grain length between Hap1 and Hap2. **(C)** Comparison of grain width between Hap1 and Hap2. **(D)** Comparison of grain thickness between Hap1 and Hap2. **(E)** Comparison of 1000-grain weight between Hap1 and Hap2. Note: WT, Wild type. Error bars indicate SEM. Statistical significance was determined by a two-tailed *t*-test (*P < 0.05, ***P < 0.001).

To explore whether natural variation in the coding region of *OsRDH* is associated with grain size and weight, we analyzed sequence polymorphisms in 533 cultivated rice accessions obtained from the Rice Genome Project database (https://ricevarmap.ncpgr.cn/vars_genotype/). Six single-nucleotide polymorphisms (SNPs) were identified within the *OsRDH* coding region, defining six major haplotypes (Hap1-Hap6) across the population (**Figure 9A**). Haplotype distribution exhibited strong subspecies differentiation, with Hap1 and Hap2 predominantly present in japonica rice, Hap3-Hap5 enriched in indica varieties, and Hap6 mainly represented in aus germplasm. Among these, Hap3 was the most frequent haplotype in the analyzed population. Using Zhonghua 11 (japonica rice, Hap1) as a reference, we compared the grain-related traits between Hap1 and Hap2 accessions. Hap2 lines exhibited a significant increase in grain length, whereas width and 1000-grain weight showed no statistically significant differences (**Figures 9B–E**). Notably, this phenotype pattern closely resembled that observed in the *Osrdh* loss-of-function mutants, which display elongated grains without a concomitant increase in individual grain mass.

### 3.10 *OsRDH* mutation alters hormone responsiveness during rice seed germination

**Figure 10:**
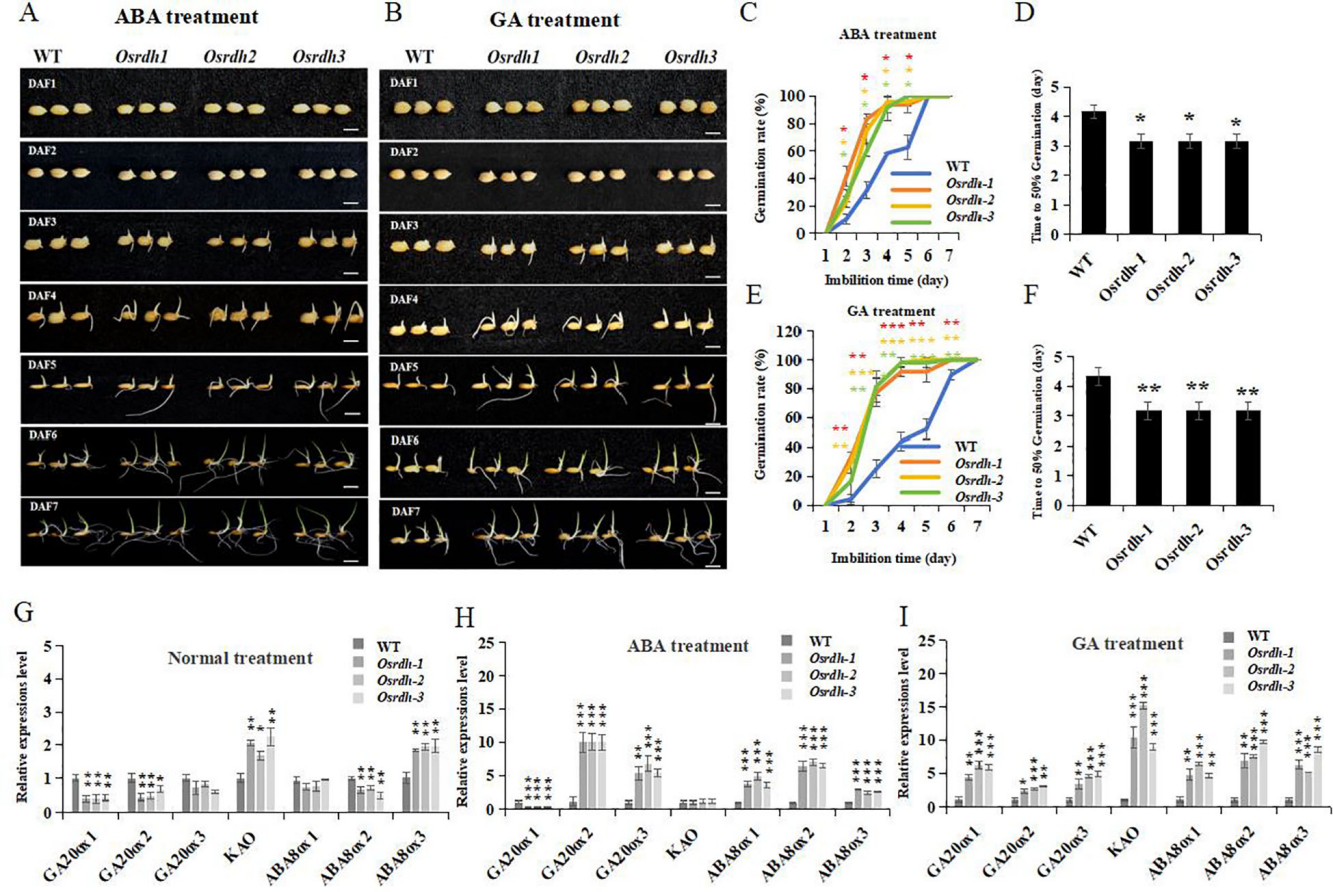
Germination and related gene expression analysis of *Osrdh* mutant seeds under ABA and GA treatment conditions. **(A)** Germination and growth of *Osrdh* mutant seeds under ABA treatment conditions, scale: 1 cm. **(B)** Germination and growth of *Osrdh* mutant seeds under GA treatment conditions, scale: 1 cm. **(C)** Statistical analysis of germination rate of *Osrdh* mutant seeds under ABA treatment conditions. **(D)** Time required for 50% germination of *Osrdh* mutant seeds under ABA treatment conditions (days). **(E)** Statistical analysis of germination rate of *Osrdh* mutant seeds under GA treatment conditions. **(F)** Time required for 50% germination of *Osrdh* mutant seeds under GA treatment conditions (days). **(G)** Expression levels of genes involved in gibberellin (GA) biosynthesis and abscisic acid (ABA) catabolism in *Osrdh* mutant seeds under normal growth conditions after 5 days. **(H)** Expression levels of genes involved in GA biosynthesis and ABA catabolism in *Osrdh* mutant seeds treated with ABA for 5 days. **(I)** Expression levels of genes involved in GA biosynthesis and ABA catabolism in *Osrdh* mutant seeds treated with GA for 5 days. WT, wild type. Each experiment was performed in triplicate for biological replication. Significant differences were determined by a two-tailed *t-*test (*P < 0.05, **P< 0.01, ***P < 0.001). Error bars represent the standard error of the mean (SEM).

Given the pronounced alterations in endogenous phytohormone levels observed in the *Osrdh* mutant and well-established roles of ABA and GA_3_ in regulating rice seed germination, we first assessed whether loss of *OsRDH* function affects germination under basal conditions. Neither the final germination rate nor the time to reach 50% germination (T50) differed significantly between the *Osrdh* mutant and the wild type (**Supplementary Figures 6A–C**), indicating that loss of *OsRDH* does not impair intrinsic seed viability. However, exogenous application of ABA (0.5 μM) or GA (5 μM) significantly increased the germination rate of *Osrdh* seeds and markedly reduced T_50_ relative to the wild type (**Figures 10A–F**), whereas treatment with IAA(5 μM) had no detectable effect (**Supplementary Figures 7A–C**). These results suggest that *Osrdh* seeds exhibit enhanced sensitivity to ABA and GA signaling rather than constitutive changes in germination capacity.

To further elucidate the molecular basis of this hormone-responsive phenotype, we examined the expression of key genes related to GA biosynthesis and ABA catabolism in seeds imbibed for 5 days under control conditions or following ABA or GA treatment. In the *Osrdh* mutant, transcripts of GA biosynthetic and metabolic genes (*OsGA20ox1*, *OsGA20ox2*, *OsGA20ox3*, and *KAO*), as well as ABA catabolic genes (*OsABAox1*, *OsABAox2*, and *OsABAox3*), were significantly upregulated in response to both ABA and GA treatments compared with normally imbibed seeds (**Figures 10G–I**). Notably, *KAO* expression was elevated in both control- and GA-treated *Osrdh* seeds, but remained unchanged under ABA treatment, suggesting a selective regulatory role of *OsRDH* in modulating GA pathway responsiveness.

Collectively, these findings indicate that *OsRDH* does not directly determine basal germination capacity but instead modulates seed sensitivity to ABA and GA, thereby fine-tuning hormone-dependent regulation of rice seed germination.

### 3.11 *OsRDH* mutation promotes primary root elongation in rice seedlings

**Figure 11:**
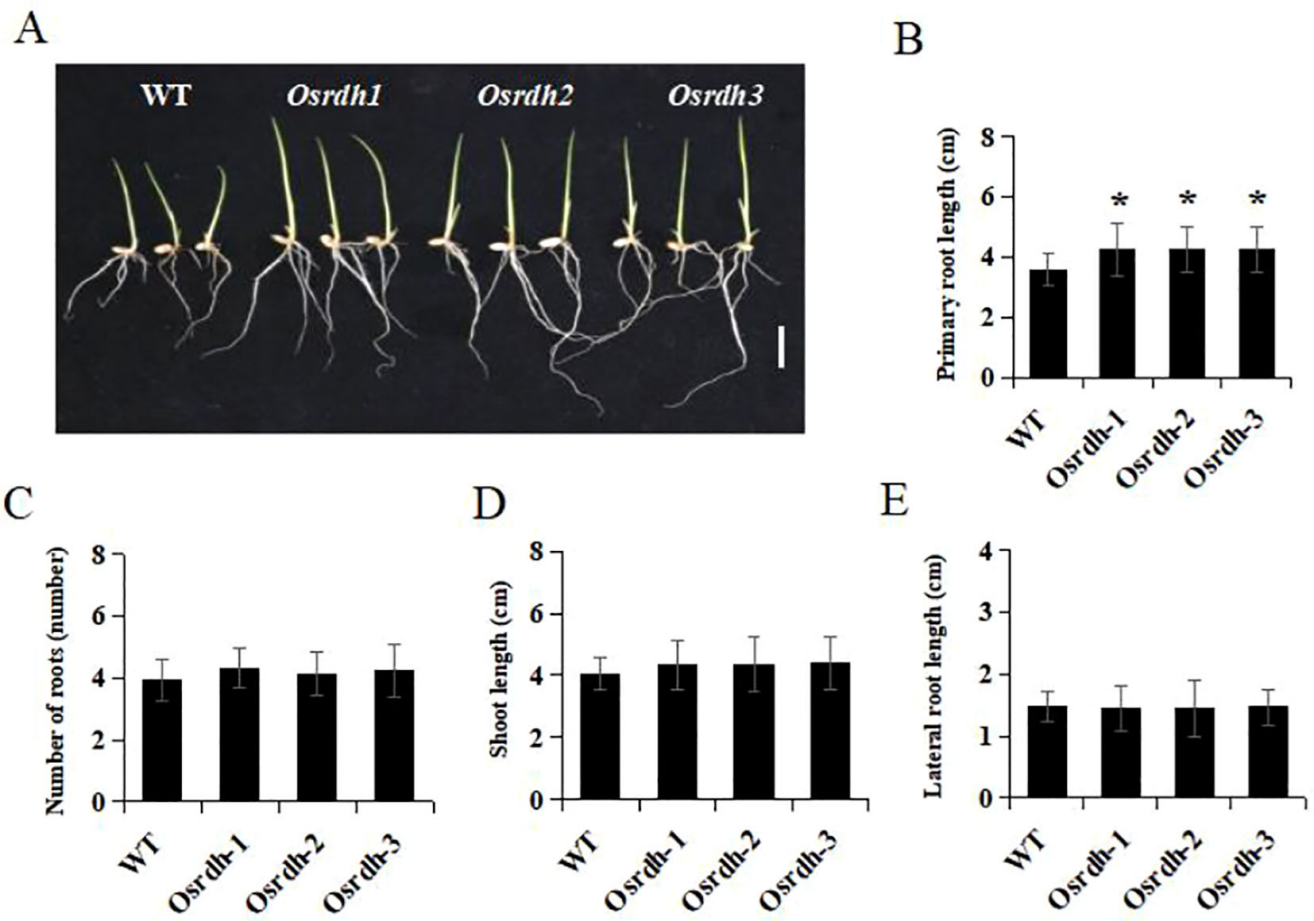
Statistical analysis of root growth in rice *Osrdh* mutant seedlings. **(A)** Statistical analysis of the main root length 7 days after germination of *Osrdh* mutant seeds, scale: 1 cm. **(B)** Root length phenotype of *Osrdh* mutant seeds 7 days after germination. **(C)** Statistical analysis of total root number in *Osrdh* mutant seeds 7 days after germination. **(D)** Statistical analysis of stem length in *Osrdh* mutant seeds 7 days after germination. **(E)** Statistical analysis of lateral root length in *Osrdh* mutant seeds 7 days after germination. Note: WT, Wild type. Each experiment was performed in triplicate for biological replication. Statistical significance was determined using a two-tailed *t-*test (*P < 0.05). Error bars represent the standard error of the mean (SEM).

Retinol dehydrogenases have been implicated in the regulation of root development in *Arabidopsis thaliana* (Dickinson et al., 2021). To investigate whether *OsRDH* plays a similar role in rice, we measured primary root length, lateral root length, total root number, and shoot length in *Osrdh* mutant seedlings at 7 days after germination. The primary root length of *Osrdh* seedlings was significantly increased compared with the wild type (**Figures 11A, B**). In contrast, no significant differences were observed in total root number, lateral root length, or shoot length (**Figures 11C–E**). These results suggest that the loss of *OsRDH* function specifically promotes primary root elongation during early seedling development, rather than broadly altering root system architecture.

## 4 Discussion

### 4.1 *OsRDH*-mediated changes in starch and protein content in rice grains

In monocotyledonous grasses, *GAPC* plays a central role in regulating plant growth, development, and responses to stress (Kim et al., 2022). Mutations in *OsGAPC3* markedly influence rice quality traits while enhancing its nutritional value (Peng et al., 2024b). With *OsRDH* expression upregulated in *Osgapc3* mutants and suppressed in *OsGAPC3* overexpressors (Peng et al., 2024b). Although retinol dehydrogenase (RDH) participates in multiple biological processes (Wu et al., 2023; Wang et al., 2022; Leung et al., 2024), its function in rice has remained unclear. Here, we show that *OsRDH* is constitutively expressed and localized to chloroplasts. Loss of *OsRDH* markedly reduces protein and amylose contents, while increasing starch and free fatty acids, highlighting its role in coordinating storage compound accumulation. Ultrastructural analysis revealed fewer PBI and PBII bodies in the mutant endosperm, accompanied by decreased albumin and glutelin, indicating that reduced protein content results from both diminished protein body formation and lower storage protein levels. These findings provide a molecular basis for improving rice nutritional quality.

Proteomic and transcriptomic analyses further revealed that downregulated proteins in the *Osrdh* endosperm are predominantly involved in amino acid, starch, and sugar metabolism. Consistently, genes related to protein biosynthesis were repressed, whereas those associated with starch synthesis were upregulated. These molecular changes align with the observed phenotypes of reduced protein and elevated starch, demonstrating that *OsRDH* regulates the balance between protein and starch accumulation in rice endosperm. Therefore, *OsRDH* is involved in regulating the expression of genes related to protein and starch synthesis metabolism, thereby affecting the accumulation of its main storage substances (**Supplementary Figure 8**). This provides important information for the genetic improvement of rice quality in the later stage.

### 4.2 *OsRDH* affects multiple rice quality traits

Grain appearance is a key determinant of rice quality and is primarily defined by grain shape, chalkiness, translucency, and color (Yang et al., 2024; Peng et al., 2014b; Peng et al., 2021). In the *Osrdh* mutant, both the chalkiness area and the degree of chalkiness were markedly reduced. Scanning electron microscopy of chalky regions revealed that starch granules were more densely packed, exhibited a uniform polygonal morphology, and were arranged more compactly. These structural features likely underlie the reduced chalkiness observed in the *Osrdh* mutant. Furthermore, Scanning Electron Microscopy of the husk of mature grains showed an increased number of outer chaff cells in the *Osrdh* mutant, which is associated with elongated grain length. These results collectively demonstrate that loss of *OsRDH* positively affects grain appearance by lowering chalkiness and enhancing grain elongation. In addition, natural allelic differences are key determinants of rice yield, grain quality variation, and subspecies divergence (Yang et al., 2025). Haplotype analysis of the *OsRDH* coding region identified six distinct haplotypes, of which Hap1 and Hap2 are predominantly distributed in *japonica* rice. Comparative analysis revealed that Zhonghua 11, which carries Hap1, exhibited significantly shorter grain length than Hap2, while no significant difference in grain width was detected. This pattern is consistent with the elongated grain phenotype of the *Osrdh* mutant, indicating that *Osrdh* allele is likely associated with Hap2. Therefore, these population-level analyses further support a close association between *OsRDH* and the genetic control of grain size.

The sensory and physicochemical aspects of rice cooking and eating quality are mainly reflected in amylose content, gel consistency, gelatinization temperature, and taste value, while processing quality is commonly measured by the rates of brown rice, milled rice, and head rice (Li et al., 2022). In the *Osrdh* mutant, amylose content was significantly reduced, while head rice yield was significantly increased. These results indicate that the mutation of *OsRDH* confers simultaneous improvements in both cooking/eating and processing quality (**Supplementary Figure 8**), highlighting its potential value for rice breeding and commercial application.

### 4.3 *OsRDH* may affect photosynthesis, thereby negatively impacting rice yield

Chloroplast abundance and photosynthetic capacity are commonly assessed by SPAD values, which reflect leaf absorbance at specific wavelengths and serve as a proxy for chlorophyll content and plant physiological status (Wang et al., 2023). Given that *OsRDH* is localized to chloroplasts, we measured pigment composition in flag leaves at the heading stage. The *Osrdh* mutant showed a marked decrease in total chlorophyll, chlorophyll a, chlorophyll b, carotenoid content, and SPAD values, suggesting a reduction in photosynthetic capacity. Because *OsRDH* expression is reportedly influenced by phytohormones (Suganami et al., 2024), we further examined endogenous hormone levels in flag leaves. The results showed significantly decreased concentrations of IAA and ABA, accompanied by a marked increase in GA3. These hormonal shifts are likely to perturb chlorophyll accumulation and photosynthetic performance, thereby linking *OsRDH* function to hormonal regulation of photosynthesis.

Grain yield is determined by multiple agronomic traits, including spikelet number per panicle, 1,000-grain weight, seed setting rate, tiller number, and yield per plant, among which spikelet number per panicle is a major determinant (Peng et al., 2024a; Yan et al., 2025). Field evaluation revealed that the *Osrdh* mutation exhibited significantly reduced plant height, fewer spikelets per panicle, and lower yield per plant. Collectively, these results indicate that the loss of *OsRDH* compromises photosynthetic capacity and associated hormonal homeostasis, ultimately exerting a negative effect on rice yield.

### 4.4 *OsRDH* mutation promotes seed germination and primary root growth

Seed germination initiates with water imbibition and culminates in radicle protrusion, a process tightly regulated by phytohormone signaling (Jia et al., 2024). Among these regulators, Gibberellins (GAs) promote plant growth, while abscisic acid (ABA) generally acts as a negative regulator of germination and stress responses (Liao et al., 2023). Germination performance, including final germination rate and the time to reach 50% germination (T_5_₀), was comparable between *Osrdh* mutant and wild-type seeds under both normal and 5 μM IAA conditions. However, under 0.5 μM ABA and 5 μM GA treatment, the *Osrdh* mutant exhibited a significantly higher germination rate and a shortened T_5_₀. Consistently, genes involved in GA biosynthesis and metabolism, as well as ABA catabolism and turnover, were significantly upregulated in mutant seeds. These results suggest that *OsRDH* modulates hormonal responsiveness, thereby enhancing GA-associated pathways and promoting ABA degradation to facilitate seed germination. In *Arabidopsis thaliana*, retinol dehydrogenase has been implicated in root development (Dickinson et al., 2021). Similarly, in rice, the *Osrdh* mutant displayed a significantly increased primary root length seven days after germination, indicating that *OsRDH* also influences early root system architecture and subsequent seedling establishment.

### 4.5 Proposed regulatory network of *OsRDH* in rice

*OsRDH* mediates a trade-off between grain quality improvement and yield through modulation of the source-sink balance. There is a universal inherent biological trade-off between the synergistic improvement of quality and yield in rice. While rice quality regulatory genes improve rice quality, changes in sink source relationships often occur, which in turn have a certain impact on the yield related traits of rice (Peng et al., 2024a, Peng et al., 2024b; Yuan et al., 2025). Our results reveal that *OsRDH* functions as a key regulator of the balance between grain quality and yield by coordinating carbon and nitrogen partitioning along the leaf-grain source-sink continuum (Lu et al., 2019; Zhang et al., 2016). Loss of *OsRDH* function led to pronounced improvements in grain appearance, processing quality, and cooking and eating quality, including reduced chalkiness, increased head rice percentage, and decreased amylose content. These favorable quality traits were accompanied by a substantial reprogramming of storage compound composition in the endosperm, characterized by enhanced starch accumulation and reduced protein and essential amino acid contents (**Supplementary Figure 8**). It is interesting that our research group found in the early stage of studying the functions of *OsAAP6* and *OsG6PGH1* genes that both of two gene affect rice quality, but do not affect rice yield (Peng et al., 2014a, Peng et al., 2024a). Therefore, the two important traits of rice yield and rice quality, the trade-off between quality and yield, are not unviable.

At the source level, the chloroplast localization of *OsRDH* and the observed reduction in chlorophyll content and SPAD values in *Osrdh* mutant flag leaves indicate impaired photosynthetic capacity. Concurrently, altered phytohormone profiles, including decreased IAA and ABA levels and increased GA_3_ levels, suggest that *OsRDH* integrates chloroplast-derived metabolic signals with hormone-mediated regulatory pathways that influence source strength and assimilate availability. At the sink level, proteomic analyses demonstrated coordinated upregulation of starch biosynthesis-related genes and enrichment of carbohydrate metabolism-associated proteins, alongside downregulation of genes and proteins involved in amino acid biosynthesis and storage protein accumulation. These molecular changes were further supported by ultrastructural evidence of reduced protein body I and II sizes and decreased accumulation of albumin and glutelin in the endosperm, collectively indicating a shift in assimilate partitioning toward preferential carbon allocation to starch at the expense of nitrogen investment in protein synthesis.

Importantly, this quality-oriented reallocation of resources is accompanied by a measurable yield penalty. Field evaluations revealed that *Osrdh* mutants exhibited reduced plant height, fewer spikelets per panicle, and lower yield per plant, while tiller number, heading date, and seed-setting rate remained largely unaffected. This pattern suggests that the disruption of source-sink coordination limits overall assimilate supply and reproductive sink capacity, thereby constraining yield potential.

Together, these findings support a model in which *OsRDH* acts as a regulatory node linking chloroplast function, and endosperm metabolic programming to fine-tune the trade-off between grain quality and yield (**Supplementary Figure 8**). Targeted modulation of *OsRDH* activity, rather than complete loss of function, may therefore represent a promising strategy for optimizing both grain quality and productivity in rice breeding programs.

## 5 Conclusion

*OsRDH* is a constitutively expressed, chloroplast-localized gene whose loss of function triggers coordinated changes in carbon and nitrogen metabolism in rice grains. In the *Osrdh* mutant, substantial alterations in starch (including amylose), protein, free fatty acids, and total essential amino acid contents collectively improve grain appearance, processing performance, and cooking/eating quality, but at the expense of yield. Moreover, *OsRDH* modulates the expression of genes involved in GA and ABA metabolism, thereby influencing seed germination and primary root growth. Together, *OsRDH* is an important quality trait gene with multiple biological functions, and is involved in various biological processes such as rice seed germination and primary root growth. Our results provide important information for the cultivation of new rice varieties with superior quality traits.

## Data Availability

The data presented in the study are deposited at https://www.cncb.ac.cn/, accession number PRJCA059147.
