# Supplementary material for: OsRDH modulates multiple grain quality traits and contributes to rice seed germination and root growth development
Source: Front Plant Sci. 2026 May 20;17:1820692. doi: 10.3389/fpls.2026.1820692 (PMC13230045; doi:10.3389/fpls.2026.1820692)
Supplement: Supplementary file 1 [file DataSheet1.docx]

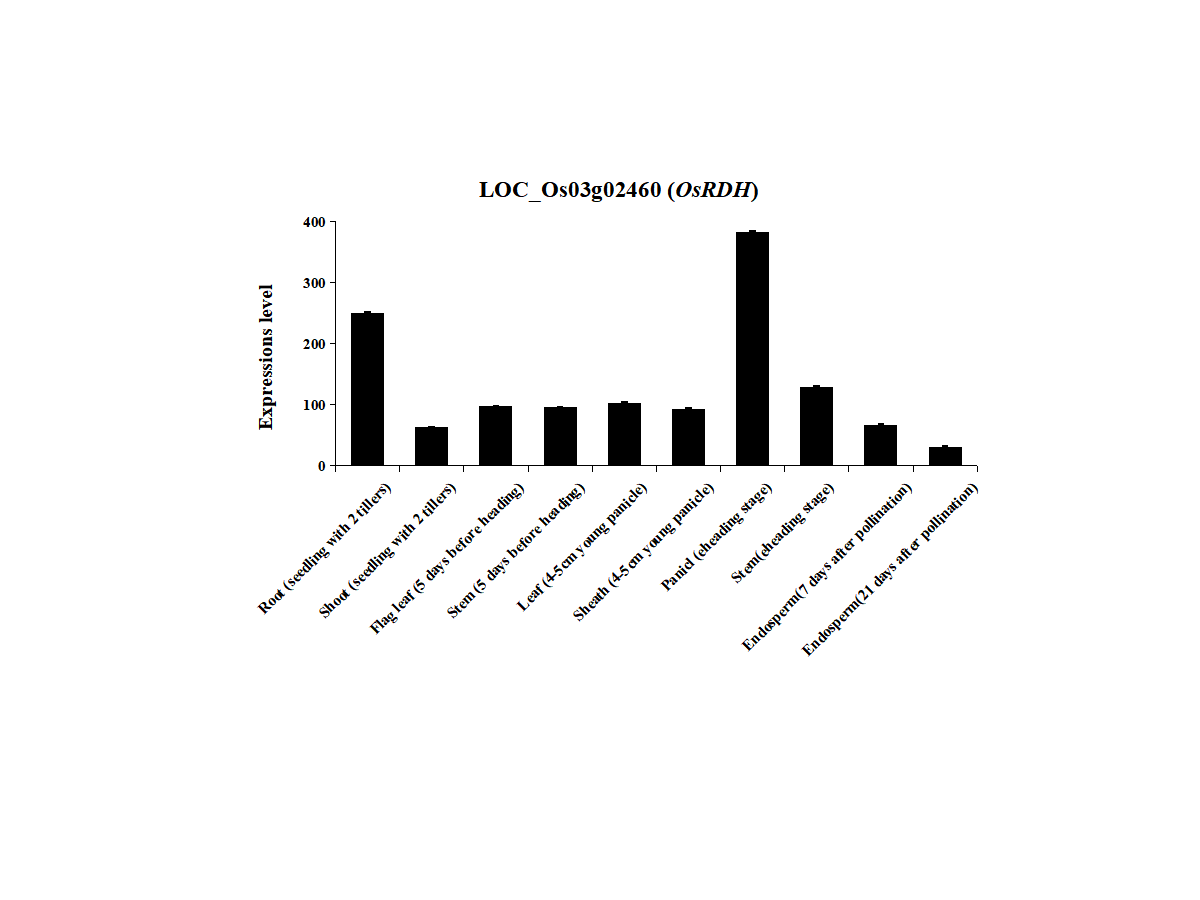


Figure S1. Analysis of expression levels of *OsRDH* (LOC_Os03g02460) in different tissues from the CREP database (<http://crep.ncpgr.cn/crep-cgi/query_by_tree.cgi>).


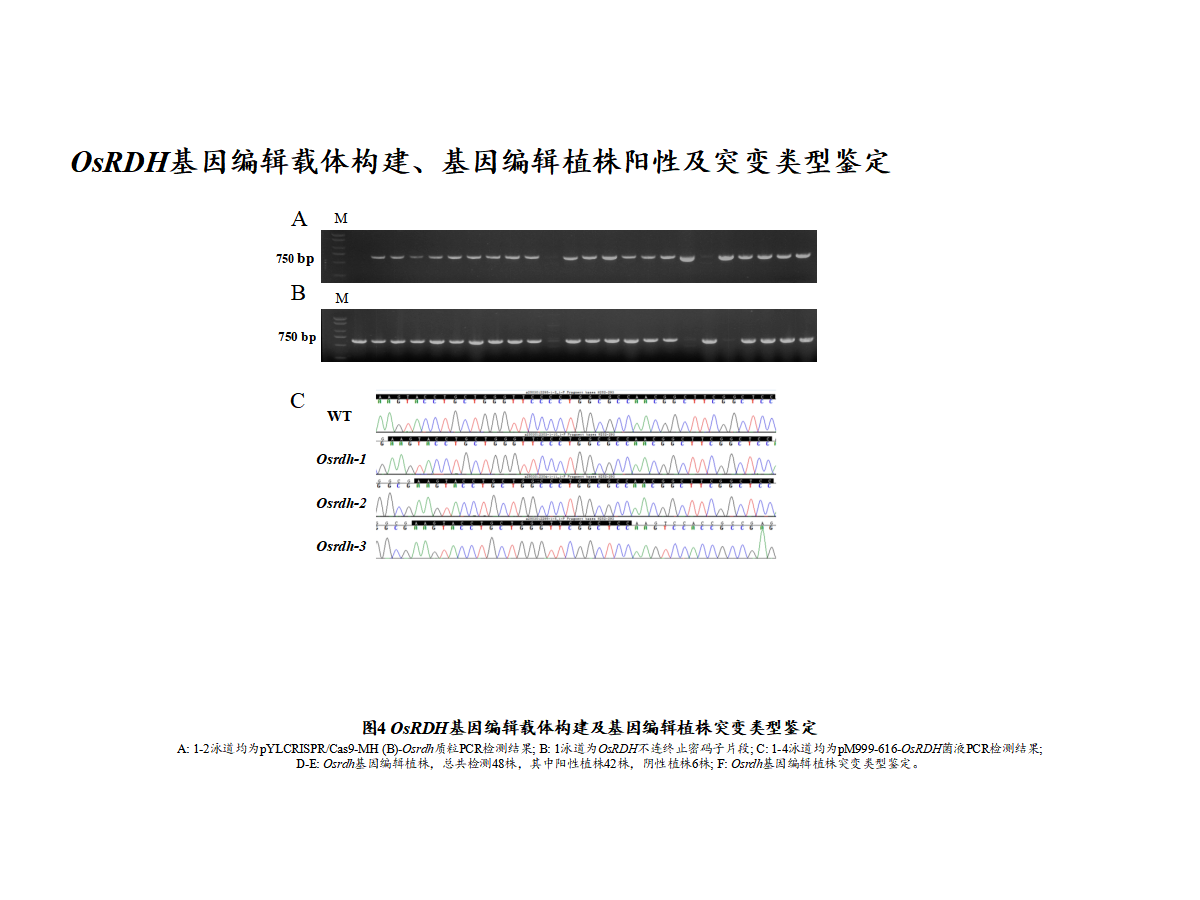


Figure S2. Identification of *Osrdh* gene-edited plants. Note: A-B: Detection of *Osrdh* gene-edited plants, with a total of 48 plants tested, including 42 positive plants and 6 negative plants; C: Sequencing peak diagram for identification of *Osrdh* mutation types. WT: Wild type.

**
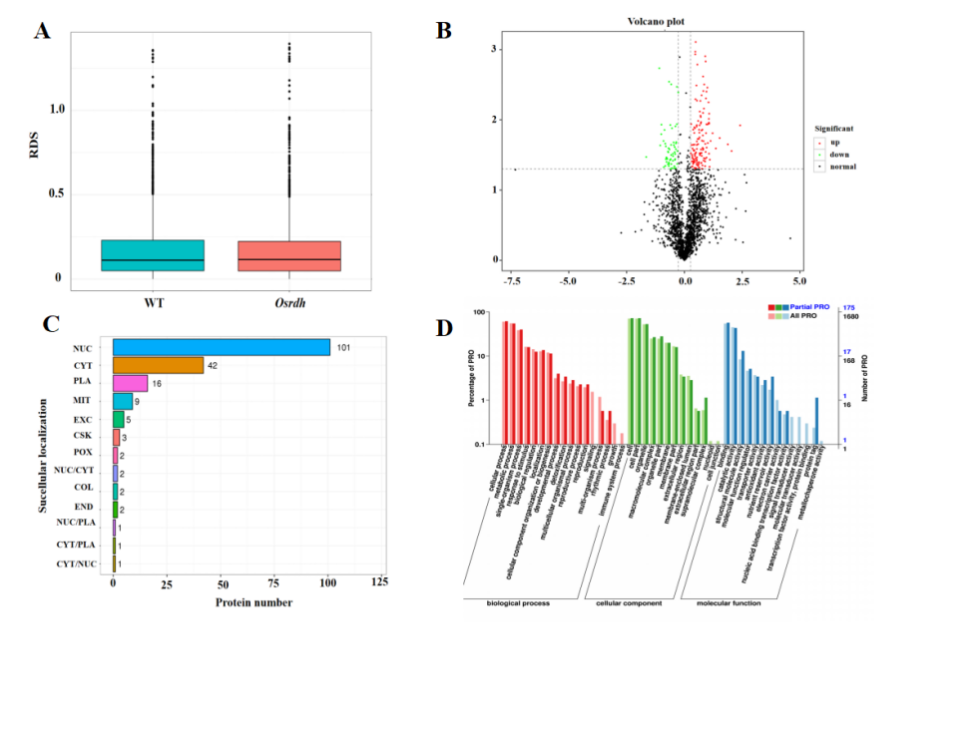
**

Figure S3. Proteomic analysis of *Osrdh* mutant endosperm. Note: A: Relative standard deviation (RSD) distribution of *Osrdh* mutant and WT groups; B: Volcano plot of DEPs in *Osrdh* mutant endosperm, with each point representing a gene; C: Subcellular localization and enrichment analysis of DEPs; D: GO database annotation and enrichment of differential proteins. WT: Wild type.


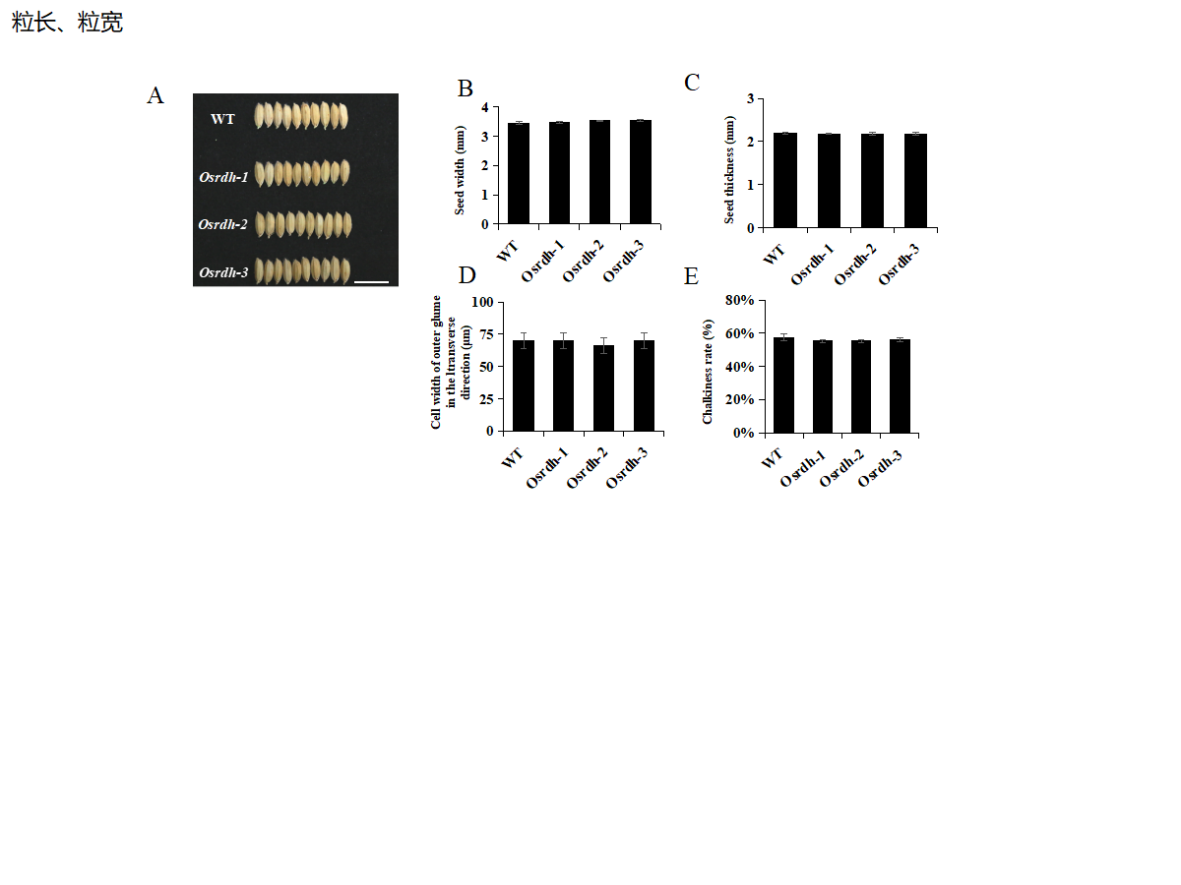


Figure S4. Detection and analysis of appearance quality traits of the *Osrdh* mutant. Note: A: Seed grain width phenotype of *Osrdh* mutant, scale: 1 cm; B: Statistical analysis of seed grain width of *Osrdh* mutant; C: Statistical analysis of seed grain thickness of *Osrdh* mutant; D: Statistical analysis of transverse length of lemma cells in seeds of *Osrdh* mutant; E: Statistical analysis of chalkiness rate of rice grains in *Osrdh* mutant. Each experiment was performed in triplicate for biological replication. Significant difference based on two-tailed *t*-test. WT: Wild type. Error bars, Standard error of the mean (SEM).


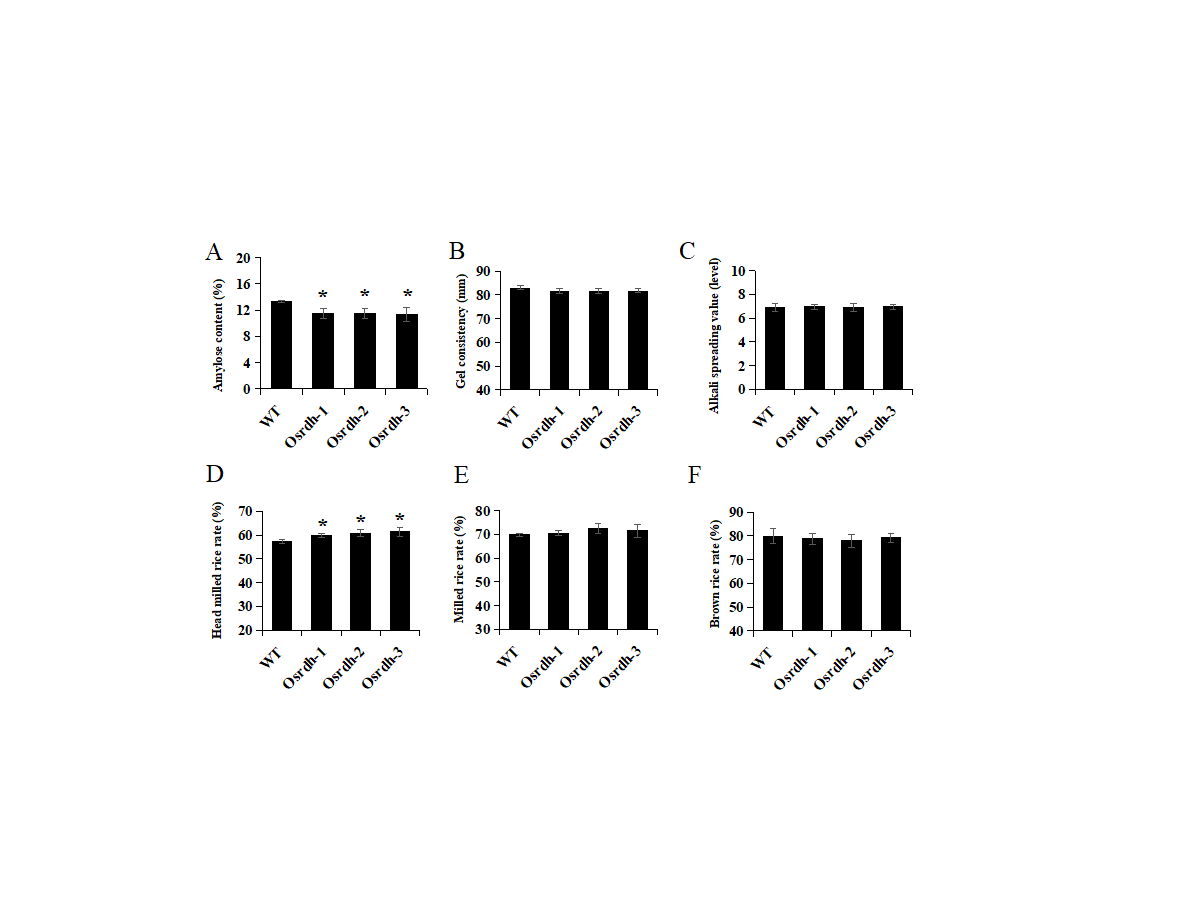


Figure S5. Detection and analysis of cooking taste and processing quality traits of *Osrdh* mutant rice. Note: A: Detection and analysis of amylose content in *Osrdh* mutant rice; B: Detection and analysis of gel consistency in *Osrdh* mutant rice; C: Detection and analysis of gelatinization temperature in *Osrdh* mutant rice; D: Statistics of head rice percentage in *Osrdh* mutant rice; E: Statistics of milled rice percentage in *Osrdh* mutant rice; F: Statistics of brown rice percentage in *Osrdh* mutant rice. Each experiment was performed in triplicate for biological replication. Significant differences were based on a two-tailed *t*-test, **P*<0.05. WT: Wild type. Error bars, Standard error of the mean (SEM).


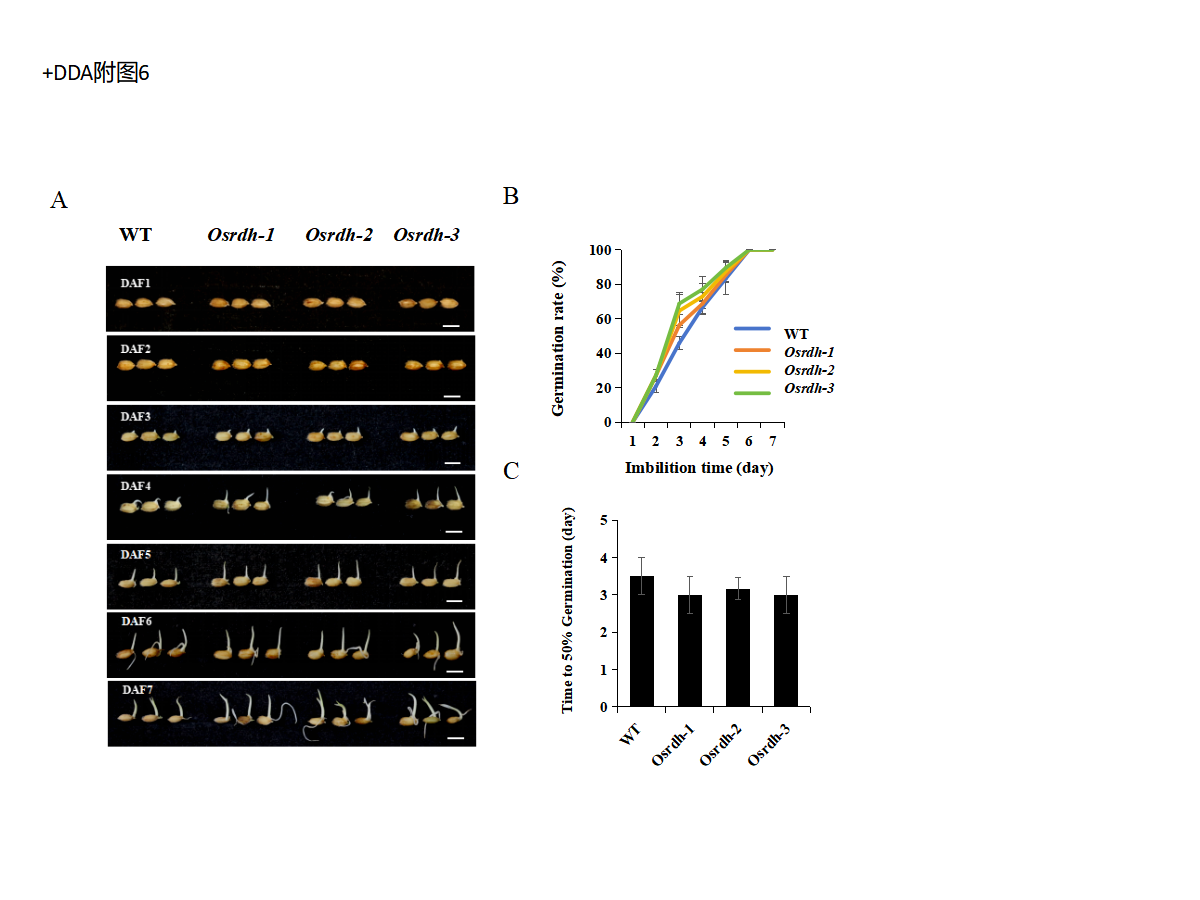


Figure S6. Germination statistics of *Osrdh* mutant seeds under normal conditions. Note: A: Phenotype of germination and growth of *Osrdh* mutant seeds, scale: 1 cm; B: Statistical analysis of germination rate of *Osrdh* mutant seeds; C: Time required for 50% germination of *Osrdh* mutant seeds (days), significant difference based on two-tailed *t*-test. WT: Wild type. Each experiment was performed in triplicate for biological replication. Error bars, Standard error of the mean (SEM).


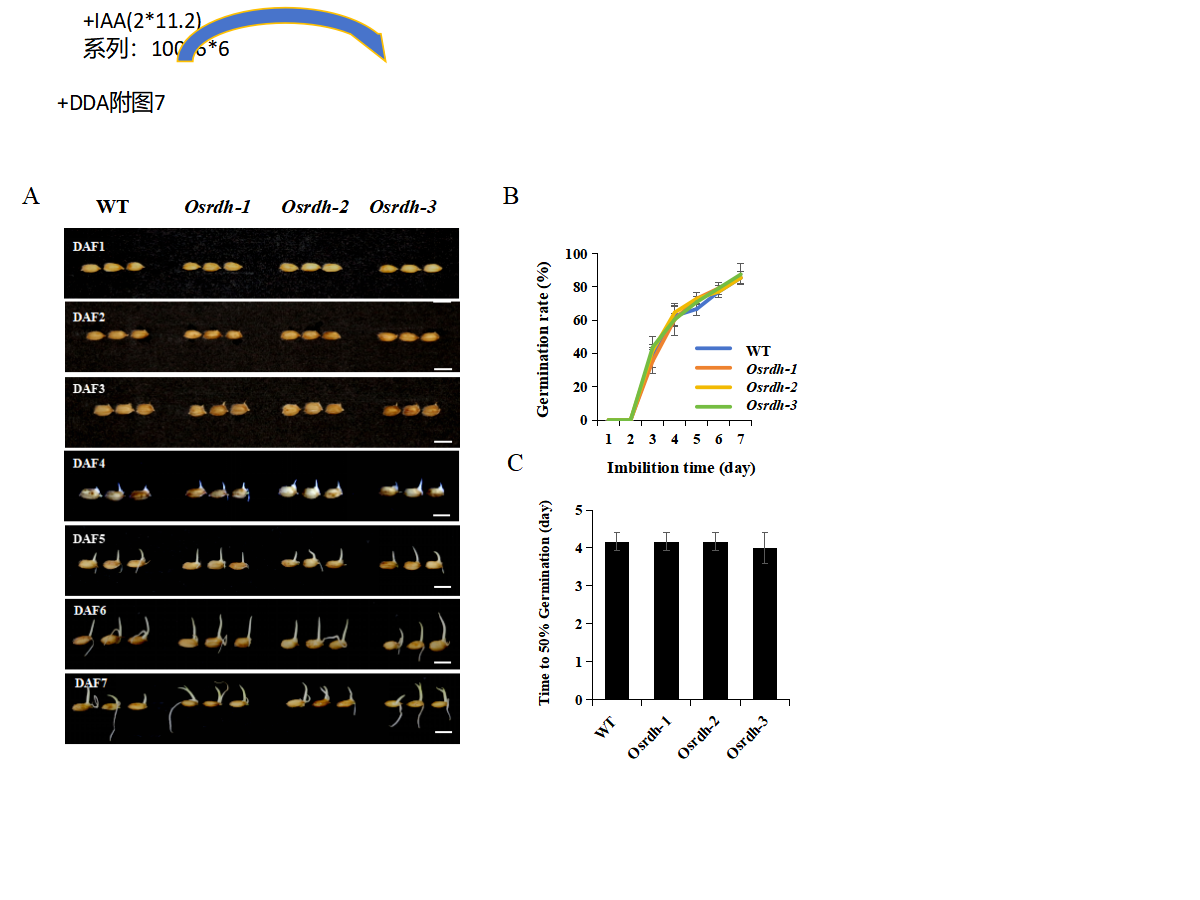


Figure S7. Germination of *Osrdh* mutant seeds under IAA treatment. Note: A: Phenotype of germination and growth of *Osrdh* mutant seeds, scale: 1 cm; B: Statistical analysis of germination rate of *Osrdh* mutant seeds; C: Time required for 50% germination of *Osrdh* mutant seeds (days); Significant differences are based on a two-tailed *t*-test. Each experiment was performed in triplicate for biological replication. WT: Wild type. Error bars, Standard error of the mean (SEM).


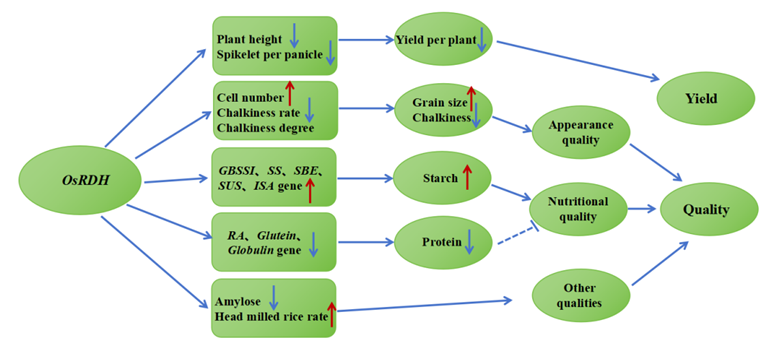


Figure S8. Proposed model of *OsRDH* function and network regulation in rice.

Table S1. Differentially expressed proteins in proteomics.

|  | Protein ID | log2 FC | *p*-value | Protein regulated |
| --- | --- | --- | --- | --- |
| 1 | A0A0N7KFT8 | -0.755919979 | 0.0352502200993281 | down |
| 2 | A0A0N7KQ01 | 0.372625478171353 | 0.0389648115357572 | up |
| 3 | A0A0P0VHP9 | 0.53947200232598 | 0.025900198136588 | up |
| 4 | A0A0P0VL75 | -0.991866405 | 0.0117028299959792 | down |
| 5 | A0A0P0VLJ4 | -0.68378039 | 0.0252780393406981 | down |
| 6 | A0A0P0VLQ3 | 0.606486096668311 | 0.0434521318937899 | up |
| 7 | A0A0P0VS15 | -0.399055276 | 0.0306270361091741 | down |
| 8 | A0A0P0VTX8 | 0.41988988962171 | 0.0275675433543403 | up |
| 9 | A0A0P0W7K6 | 0.85733310977389 | 0.00313521561395009 | up |
| 10 | A0A0P0WFP9 | 0.961436294059916 | 0.0248443739988706 | up |
| 11 | A0A0P0XU67 | 0.544074127865924 | 0.00531960821132732 | up |
| 12 | A0A0P0XUE4 | 0.62405696990168 | 0.0475842184506749 | up |
| 13 | A0A0P0Y1Y5 | 0.741903096851598 | 0.0296234909701074 | up |
| 14 | A3AB40 | -1.084968281 | 0.00183629414732903 | down |
| 15 | A3C6D7 | 0.448506869908836 | 0.0399298442692713 | up |
| 16 | B7E390 | 1.01094018526523 | 0.0213973534421058 | up |
| 17 | B7EME6 | 0.656597002977422 | 0.0298933489878482 | up |
| 18 | B9FHF3 | -0.431741782 | 0.0456174318383145 | down |
| 19 | C7J0T2 | 0.472179246189363 | 0.00106973530936421 | up |
| 20 | O65091 | 1.06561345480774 | 0.0302642296468683 | up |
| 21 | P07730 | 0.435812005114064 | 0.0225107748513263 | up |
| 22 | P0DKK1 | -0.492234069 | 0.0328802016917228 | down |
| 23 | P0DKK3 | 0.432333243077166 | 0.0393365013006442 | up |
| 24 | P0DKK7 | 0.777821767014345 | 0.0138008313695379 | up |
| 25 | P0DKK8 | 0.807717930461652 | 0.0262620934511511 | up |
| 26 | P17784 | 0.426094252981098 | 0.0153629121786169 | up |
| 27 | P29421 | 0.432018076993235 | 0.0483664934748747 | up |
| 28 | P35683 | 0.672458227946009 | 0.0305328980366374 | up |
| 29 | P37833 | 0.733816532139047 | 0.0408056526474225 | up |
| 30 | P49027 | 0.670921669773312 | 0.0317912969284798 | up |
| 31 | P49397 | 0.340877671522989 | 0.0314523696754841 | up |
| 32 | P51431 | 0.385382659692337 | 0.0409537228932318 | up |
| 33 | P51823 | 0.805539888803461 | 0.00241056975098681 | up |
| 34 | P55142 | -0.453933627 | 0.0228652054559861 | down |
| 35 | Q01881 | 0.346157281495691 | 0.0459370038632359 | up |
| 36 | Q06398 | 0.830063912159737 | 0.0379515199410652 | up |
| 37 | Q09151 | 0.429962045763076 | 0.0127911104484722 | up |
| 38 | Q0D3N0 | -1.649919875 | 0.0337667901627309 | down |
| 39 | Q0D9C4 | 0.48469814880652 | 0.0351700597943063 | up |
| 40 | Q0DDE3 | 0.952344576452748 | 0.0115994217541115 | up |
| 41 | Q0DEC8 | 0.38706605047712 | 0.0362843263080307 | up |
| 42 | Q0DEV5 | 0.654099004676661 | 0.017762074747606 | up |
| 43 | Q0DIR7 | 0.895260298895494 | 0.0391555617058611 | up |
| 44 | Q0DKM4 | -0.825760418 | 0.0217950390583161 | down |
| 45 | Q0DYB1 | -0.999062 | 0.016015352107412 | down |
| 46 | Q0E2C9 | -0.464554969 | 0.0209631201120287 | down |
| 47 | Q0IM09 | 0.399641135243688 | 0.0402193310713559 | up |
| 48 | Q0IQK9 | 0.642915657189741 | 0.0479773957051338 | up |
| 49 | Q0ITS8 | 0.998716492168772 | 0.0116017935638745 | up |
| 50 | Q0J0W2 | -1.043035327 | 0.0231247857451546 | down |
| 51 | Q0J5G1 | 0.843559319554013 | 0.0350931197134671 | up |
| 52 | Q0J9Y2 | 0.35096491004792 | 0.0255831316271753 | up |
| 53 | Q0JCA4 | 0.503342602808499 | 0.0291276020917267 | up |
| 54 | Q0JCB3 | -0.713599759 | 0.0428379601714507 | down |
| 55 | Q0JDA2 | -0.419429261 | 0.0244364064977481 | down |
| 56 | Q0JDN0 | 0.593942309095047 | 0.0103462959130026 | up |
| 57 | Q0JEK1 | 0.902464963703673 | 0.00124237748998363 | up |
| 58 | Q0JNL7 | -0.326404496 | 0.00338019667827137 | down |
| 59 | Q0JNR2 | -0.411167674 | 0.0370791279589218 | down |
| 60 | Q0JQT3 | 0.53038298383549 | 0.0382095817089017 | up |
| 61 | Q0JR02 | 0.533218997531339 | 0.0119057535646089 | up |
| 62 | Q109R6 | -0.675867066 | 0.045425202988354 | down |
| 63 | Q10MQ2 | 0.919971977180401 | 0.0014737593513367 | up |
| 64 | Q10MW3 | 0.575936268327524 | 0.018690526709044 | up |
| 65 | Q10NQ3 | 0.895897614845713 | 0.0455382090415405 | up |
| 66 | Q10P20 | -0.779124187 | 0.0342646367478104 | down |
| 67 | Q10P60 | -0.802516719 | 0.036329213613293 | down |
| 68 | Q10RP0 | 0.52789107045527 | 0.0433378943514047 | up |
| 69 | Q2QN58 | 1.35788822602469 | 0.0255233706224904 | up |
| 70 | Q2QNZ3 | -0.49733295 | 0.0303559886466887 | down |
| 71 | Q2QQ53 | -0.89151979 | 0.047215516594887 | down |
| 72 | Q2QVC1 | 0.750611380435247 | 0.0326462245911304 | up |
| 73 | Q2QXQ7 | -0.744017737 | 0.0361960891255272 | down |
| 74 | Q2R1J8 | 0.743185586337409 | 0.01413249159639 | up |
| 75 | Q2R480 | 0.646600803784099 | 0.015228456934387 | up |
| 76 | Q2R8Z5 | 0.521384118488324 | 0.00323501120657817 | up |
| 77 | Q2RAK2 | 0.561135239200243 | 0.00163037711655672 | up |
| 78 | Q43009 | 0.545860232615205 | 0.0341953407110421 | up |
| 79 | Q53JG0 | 1.08923884321117 | 0.0465523545918113 | up |
| 80 | Q5JNJ5 | -0.675471902 | 0.040347373149537 | down |
| 81 | Q5N9F4 | 0.744031978877166 | 0.00446236718026424 | up |
| 82 | Q5QM60 | 0.936353178847978 | 0.0154771940633703 | up |
| 83 | Q5SNJ4 | 0.655310009517786 | 0.0119513568431835 | up |
| 84 | Q5VRX8 | 1.06027668776261 | 0.0109769473519071 | up |
| 85 | Q5W6H1 | 0.622201664155424 | 0.0186890318647532 | up |
| 86 | Q5WA72 | 0.393623400175277 | 0.0271252644102248 | up |
| 87 | Q5WMR0 | 0.785841092195283 | 0.025066324483427 | up |
| 88 | Q5Z402 | -0.803210685 | 0.0238533692086829 | down |
| 89 | Q5Z6P9 | -0.353005591 | 0.0207716175705518 | down |
| 90 | Q5Z9H7 | -0.82652098 | 0.0452474800668279 | down |
| 91 | Q5Z9Z3 | -0.537493309 | 0.0131858620927873 | down |
| 92 | Q655T1 | 0.377106695573855 | 0.0423810786558416 | up |
| 93 | Q65XA0 | -0.859096466 | 0.0139181411681673 | down |
| 94 | Q65XA1 | 0.380835167457709 | 0.0226516036938781 | up |
| 95 | Q65XK0 | 0.428248675421994 | 0.0270155180228972 | up |
| 96 | Q65XV6 | 1.10131061056625 | 0.0369541502016027 | up |
| 97 | Q67UF5 | -0.401138043 | 0.0362105369240144 | down |
| 98 | Q69IN8 | -0.660263628 | 0.0425027440521109 | down |
| 99 | Q69QD5 | -0.422164137 | 0.0288301457910287 | down |
| 100 | Q69Y99 | -0.500016809 | 0.0382307600181427 | down |
| 101 | Q6ASY2 | 1.04343883721391 | 0.00563329424089933 | up |
| 102 | Q6AVA8 | 0.901737497099626 | 0.0358362278163874 | up |
| 103 | Q6AVT2 | 0.804029819858505 | 0.00795507425795745 | up |
| 104 | Q6ES11 | 0.722509748684722 | 0.0107716096218183 | up |
| 105 | Q6ESB6 | -0.643021016 | 0.0263573748953932 | down |
| 106 | Q6ESW6 | 0.321840654484681 | 0.0390227831110813 | up |
| 107 | Q6ETQ8 | 0.29337139422685 | 0.0115069142757721 | up |
| 108 | Q6F2Y7 | 0.583955498486735 | 0.0449203089291626 | up |
| 109 | Q6H4L2 | 0.708209755333074 | 0.00909327180259398 | up |
| 110 | Q6H547 | 0.93301134021692 | 0.0049517353355829 | up |
| 111 | Q6H6C7 | 0.5278868143486 | 0.0124642388445704 | up |
| 112 | Q6H7M1 | 0.81320400748809 | 0.0257557074819222 | up |
| 113 | Q6K4S7 | -0.661730181 | 0.00286080955381228 | down |
| 114 | Q6K508 | 0.379013648673203 | 0.0137835967637799 | up |
| 115 | Q6K5G8 | 0.665914067554661 | 0.00762003567024644 | up |
| 116 | Q6K5R6 | 0.708760661746611 | 0.0112563068420214 | up |
| 117 | Q6K667 | 0.955531093838525 | 0.00396071902839186 | up |
| 118 | Q6K683 | 0.456855768386445 | 0.032710942458049 | up |
| 119 | Q6K853 | 1.06894247286408 | 0.0112981330023143 | up |
| 120 | Q6YS69 | 0.715368463923504 | 0.0264299587677784 | up |
| 121 | Q6YSB2 | 0.47725610036492 | 0.0300377964862791 | up |
| 122 | Q6YSG9 | 2.04122405766023 | 0.0278431333439596 | up |
| 123 | Q6YYV1 | 0.621876072497396 | 0.0413927897720094 | up |
| 124 | Q6YZH8 | 0.5393968674334 | 0.0202967780635331 | up |
| 125 | Q6Z387 | -0.620919912 | 0.0374270254566015 | down |
| 126 | Q6Z3A3 | 0.624852312301906 | 0.0399277875769047 | up |
| 127 | Q6Z549 | -0.902420841 | 0.0199893658695835 | down |
| 128 | Q6Z6G8 | 0.538344974516516 | 0.0227457794322689 | up |
| 129 | Q6Z744 | 0.343765281333217 | 0.0334340322939039 | up |
| 130 | Q6Z782 | 1.52596322192445 | 0.0181945576339284 | up |
| 131 | Q6Z7V2 | -0.579191668 | 0.0227031001234688 | down |
| 132 | Q6ZBH2 | 0.500057044416083 | 0.0484656402365654 | up |
| 133 | Q6ZFJ0 | -0.571994874 | 0.00312341426243715 | down |
| 134 | Q6ZGU9 | 0.467501084122167 | 0.0493530249644805 | up |
| 135 | Q6ZHC3 | 0.706282813913885 | 0.0296703559391869 | up |
| 136 | Q6ZJI3 | 0.795899562802804 | 0.0166489136087413 | up |
| 137 | Q75HJ3 | 0.312021867719473 | 0.0227660564044897 | up |
| 138 | Q75HX0 | 0.27489919204536 | 0.029876539194027 | up |
| 139 | Q75J18 | 0.447989283447187 | 0.0308891541914155 | up |
| 140 | Q75M67 | -0.574173988 | 0.0316937390269304 | down |
| 141 | Q75U53 | 1.0495416565371 | 0.0112203671202706 | up |
| 142 | Q762B4 | 0.757312415159886 | 0.0197868053966759 | up |
| 143 | Q7FAM9 | -0.710007531 | 0.0283329968799077 | down |
| 144 | Q7G065 | 0.903454288564922 | 0.0219800365085801 | up |
| 145 | Q7X8R5 | -0.696270627 | 0.0289517771237851 | down |
| 146 | Q7XD74 | -0.592218848 | 0.0362160901478702 | down |
| 147 | Q7XHV1 | -0.60729997 | 0.0118390230525395 | down |
| 148 | Q7XKI5 | 0.781285035911136 | 0.044503688566313 | up |
| 149 | Q7XLP7 | 0.544150111392373 | 0.0408339814252384 | up |
| 150 | Q7XLX6 | 1.1341482847826 | 0.019983673919767 | up |
| 151 | Q7XLZ7 | -0.45106297 | 0.0486842078888037 | down |
| 152 | Q7XQP9 | -0.565231888 | 0.0426191698763026 | down |
| 153 | Q7XUG1 | 0.992762262608416 | 0.00347489691881867 | up |
| 154 | Q7XUW5 | -0.370966404 | 0.0261120942129462 | down |
| 155 | Q84M35 | 0.797091827142412 | 0.026697295671135 | up |
| 156 | Q84M59 | 1.21935817785318 | 0.0155194945382698 | up |
| 157 | Q84P96 | 0.918965376600325 | 0.0286308485966197 | up |
| 158 | Q84Q83 | 1.05779072844153 | 0.00967336637727066 | up |
| 159 | Q84TX6 | -0.301652625 | 0.0278695950359524 | down |
| 160 | Q84VG0 | -0.327326932 | 0.011549299437638 | down |
| 161 | Q851J9 | 1.07172423847972 | 0.00815422678672711 | up |
| 162 | Q8H3I3 | 0.478248515196295 | 0.00506975307436708 | up |
| 163 | Q8H4M5 | -0.365368634 | 0.0216108934388398 | down |
| 164 | Q8H590 | 0.536732060851192 | 0.0367636163196418 | up |
| 165 | Q8H5N9 | 0.353442258995275 | 0.0352903706728121 | up |
| 166 | Q8H7U1 | 0.826680723082785 | 0.0201207182269374 | up |
| 167 | Q8H858 | -0.599540153 | 0.0499856938092585 | down |
| 168 | Q8H8U5 | -0.389772638 | 0.0123412939570954 | down |
| 169 | Q8H920 | 0.818735726192537 | 0.00388175147410511 | up |
| 170 | Q8L4L4 | 0.633117062283969 | 0.0138190697124877 | up |
| 171 | Q8LJU5 | 0.731688867917751 | 0.0413153261638537 | up |
| 172 | Q8LQ33 | 0.59269376021653 | 0.0221369436049775 | up |
| 173 | Q8RZU9 | -0.31098111 | 0.0423520730505368 | down |
| 174 | Q8RZW7 | 0.843330144908112 | 0.00690068083041278 | up |
| 175 | Q8S0J7 | -0.263247349 | 0.00406839415941959 | down |
| 176 | Q8S5M6 | 2.41133741202365 | 0.0119888694998397 | up |
| 177 | Q8S9Z3 | -0.772604986 | 0.0396371471839463 | down |
| 178 | Q8W1L6 | 0.691944107039724 | 0.0053669680714116 | up |
| 179 | Q93X08 | 0.45214748429067 | 0.0131533150143933 | up |
| 180 | Q94DE9 | 0.442214668358372 | 0.0373654649454502 | up |
| 181 | Q94E63 | -0.376523457 | 0.0485746707809235 | down |
| 182 | Q94GQ6 | -0.54171799 | 0.0487426943416315 | down |
| 183 | Q9ASJ1 | 0.956907006720343 | 0.0344304497687553 | up |
| 184 | Q9AUQ4 | 0.333048986046319 | 0.0463925301923545 | up |
| 185 | Q9LD82 | 0.488058347246825 | 0.000777059611716288 | up |
| 186 | Q9SDG5 | 0.698628177033926 | 0.00874749098336486 | up |
| 187 | Q9SDK4 | 0.46993801562674 | 0.00116266334813465 | up |
| 188 | cRAP-P02768 | 1.87998842648387 | 0.0223130563132181 | up |
| 189 | cRAP-P02769 | 0.76184611746843 | 0.0496784108050256 | up |

Table S2. Differentially expressed proteins related to plant growth and development in starch and protein metabolism.

|  | Protein ID | Gene name | Gene ID | Function | Protein regulated |
| --- | --- | --- | --- | --- | --- |
| 1 | A0A0P0VS15 | *OsBTF3* | Os03g0109600 | Affects pollen development | down |
| 2 | Q0JCB3 | *OsPIMT2* | Os04g0481400 | Involved in repairing protein damage in cells | down |
| 3 | Q10P60 | *OsHSP26.7* | Os03g0245800 | Affects plant development | down |
| 4 | Q65XA0 | *OsDHAR1* | Os05g0116100 | Affects rice yield | down |
| 5 | Q67UF5 | *OsPDIL2* | Os09g0451500 | Involved in starch and protein biosynthesis | down |
| 6 | Q84TX6 | *OsPHY2* | Os03g0818100 | Involved in seed development process | down |
| 7 | Q94E63 | *OsAlba1* | Os01g0173100 | May affects grain filling | down |
| 8 | A0A0P0W7K6 | *OsPUL3* | Os04g0164900 | Involved in starch biosynthesis | up |
| 9 | A0A0P0XU67 | *FLO12* | Os10g0390500 | Involved in starch and protein biosynthesis affecting rice quality | up |
| 10 | P07730 | *OsGluA2* | Os10g0400200 | Involved in starch and protein biosynthesis | up |
| 11 | Q0JEK1 | *OsDHDPS* | [Os04g0254000](https://rapdb.dna.affrc.go.jp/locus/?name=Os04g0254000) | Involved in protein biosynthesis | up |
| 12 | Q10MW3 | *Ospdc2* | [Os03g0262900](https://rapdb.dna.affrc.go.jp/locus/?name=Os03g0262900) | Involved in starch and protein biosynthesis | up |
| 13 | Q10RP0 | *OsCDC48* | Os03g0151800 | Involved in protein biosynthesis | up |
| 14 | Q2RAK2 | *OsPK1* | Os11g0148500 | Affects grain filling | up |
| 15 | Q6K5G8 | *OsGAPC3* | Os02g0601300 | Involved in starch and protein biosynthesis | up |
| 16 | Q6Z782 | *OsBT1* | Os02g0202400 | Involved in starch biosynthesis | up |
| 17 | Q7G065 | *OsAGPL2* | Os01g0633100 | Involved in starch and protein biosynthesis | up |
| 18 | Q94DE9 | *SBDCP1* | Os01g0856900 | Involved in starch biosynthesis | up |

Table S3. Primer sequences of protein-related genes.

| Name | Primer sequence(5'to3') |
| --- | --- |
| *RA16-F* | AGGTAGTGATCTCGGCGTTG |
| *RA16-R* | CCGATTCCTGGCTGACATAG |
| *GluteinA1-F* | CATTTGAGCCAATTCGGAGT |
| *GluteinA1-R* | GGCCTGATTGTTGGAACTGT |
| *GluteinA2-F* | GCAAGAGCAGGAACAAGGAC |
| *GluteinA2-R* | CCTCATGGTGCAAAAGGTCT |
| *GluteinA3-F* | TGAAAACCAACCCTGACTCC |
| *GluteinA3-R* | ACTCATCTCCCCTCTTGTGC |
| *GluteinB4-F* | GCGACCAGAAGGCTACAAAG |
| *GluteinB4-R* | TTGCTTGTTGATCGTTGCTC |
| *10KD Prolamin-F* | TGCAGTATTTCCCACCAACA |
| *10KD Prolamin-R* | ACATGAACATGGCTGTGGAG |
| *17KD Prolamin-F* | TTTGATGCTTGCACCTATGG |
| *17KD Prolamin-R* | GCAGCTGCTCAGTTTTAGCC |
| *Globulin1-F* | TTCTCGGTATTGCTCCTCGT |
| *Globulin1-R* | CTTATTCCTGGCCGACATTG |
| *RA17-F* | ATCGAGAACGGCGAGAAGT |
| *RA17-R* | GGACGGAGATGGTATGGAGA |
| *11S Globulin-F* | CACCAAACCCGATCTTCAGT |
| *11S Globulin-R* | CGGAACAGCTTCTCCATCTC |
| *β-actin-F* | TGCTATGTACGTCGCCATCCAG |
| *β-actin-R* | AATGAGTAACCACGCTCCGTCA |

Table S4. Primer sequences of starch-related genes.

| Gene | Primer sequence(5'to3') |
| --- | --- |
| *GBSSI-F* | TCCGAGAGGTTCAGGTCATC |
| *GBSSI-R* | ATGAGCTCCTCGGCGTAGTA |
| *SSI-F* | TCATGGATGTGAAGGAGCAA |
| *SSI-R* | TGGCAGTGAACCACAAACAT |
| *SSIIa-F* | GATCGACCAGGATGACGATT |
| *SSIIa-R* | GGGTAAAGCACCTGCAACAT |
| *SBE-F* | GGCATTGCACTCCAAAAGAT |
| *SBE-R* | GCTCCAGTTGTTGCCTTCTC |
| *Susy3-F* | CATGTACCCCCTGCTCAACT |
| *Susy3-R* | GTCAGCTGTAATGCCTGCAA |
| *SSIVa-F* | GGGAGCGGCTCAAACATAAA |
| *SSIVa-R* | CCGTGCACTGACTGCAAAAT |
| *ISA2-F* | TAGAGGTCCTCTTGGAGG |
| *ISA2-R* | AATCAGCTTCTGAGTCACCG |
| *SBEIIa-F* | GCCAATGCCAGGAAGATGA |
| *SBEIIa-R* | TGGCGACCATAGCTTTCT |
| *ISA1-F* | TGCTCAGCTACTCCTCCATCATC |
| *ISA1-R* | AGGACCGCACAACTTCAACATA |
